# Set-Up and Validation of a High Throughput Screening Method for Human Monoacylglycerol Lipase (MAGL) Based on a New Red Fluorescent Probe

**DOI:** 10.3390/molecules24122241

**Published:** 2019-06-15

**Authors:** Matteo Miceli, Silvana Casati, Roberta Ottria, Simone Di Leo, Ivano Eberini, Luca Palazzolo, Chiara Parravicini, Pierangela Ciuffreda

**Affiliations:** 1 Dipartimento di Scienze Biomediche e Cliniche “Luigi Sacco”, Università degli Studi di Milano, Via G.B. Grassi 74, 20157 Milano, Italy; matteo.miceli@unimi.it (M.M.); silvana.casati@unimi.it (S.C.); roberta.ottria@unimi.it (R.O.); 2 Dipartimento di Biotecnologie Mediche e Medicina Traslazionale, Università degli Studi di Milano, Via Fratelli Cervi 93, 20090 Segrate (MI), Italy; simone.dileo@unimi.it; 3 Dipartimento di Scienze Farmacologiche e Biomolecolari, Università degli Studi di Milano, Via Balzaretti 9, 20133 Milano, Italy; ivano.eberini@unimi.it (I.E.); luca.palazzolo@unimi.it (L.P.); chiara.parravicini@unimi.it (C.P.)

**Keywords:** high-throughput screening, endocannabinoid, monoacylglycerol lipase, assay development

## Abstract

Monoacylglycerol lipase (MAGL) is a serine hydrolase that has a key regulatory role in controlling the levels of 2-arachidonoylglycerol (2-AG), the main signaling molecule in the endocannabinoid system. Identification of selective modulators of MAGL enables both to provide new tools for investigating pathophysiological roles of 2-AG, and to discover new lead compounds for drug design. The development of sensitive and reliable methods is crucial to evaluate this modulatory activity. In the current study, we report readily synthesized long-wavelength putative fluorogenic substrates with different acylic side chains to find a new probe for MAGL activity. 7-Hydroxyresorufinyl octanoate proved to be the best substrate thanks to the highest rate of hydrolysis and the best Km and Vmax values. In addition, in silico evaluation of substrates interaction with the active site of MAGL confirms octanoate resorufine derivative as the molecule of choice. The well-known MAGL inhibitors URB602 and methyl arachidonylfluorophosphonate (MAFP) were used for the assay validation. The assay was highly reproducible with an overall average Z′ value of 0.86. The fast, sensitive and accurate method described in this study is suitable for low-cost high-throughput screening (HTS) of MAGL modulators and is a powerful new tool for studying MAGL activity.

## 1. Introduction

The endocannabinoid system (ES) is a modulatory system that plays a critical role in a variety of neuronal functions including motor activity, nociception, and appetite control as well as peripheral functions such as energy metabolism and inflammation [1]. This wide range of effects makes it an interesting target for drug development although a difficult one to act upon due to life-threatening psychological adverse effects [2] linked to the direct antagonist action against the endocannabinoid receptors.

Inhibition or enhancement of the ES hydrolytic regulatory enzymes seems a very promising strategy to act on the ES in a more fine-tuned way [3]. Monoacylglycerol lipase (MAGL), a soluble serine hydrolase that associates with cell membranes, is a very promising target for pharmacological inhibition, since it is the main hydrolase implicated in the degradation of the main signaling molecule in the ES, 2-AG. The hydrolysis of 2-AG is catalyzed also by two additional serine hydrolases, α-β hydrolase domain 6 and 12 (ABHD6 and ABHD12). MAGL, however, is responsible for approximately 85% of 2-AG hydrolysis [4]. 

Identification of new promising lead compounds for MAGL inhibition from large libraries of molecules needs simple, highly sensitive, specific, inexpensive and reliable assays suitable for a high-throughput format. The most commonly assay procedures used to screen and characterize MAGL inhibitors quantify the hydrolysis products of radiolabeled MAGL substrates [5,6] or the arachidonic acid released upon 2-AG hydrolysis by using high-performance liquid chromatography (HPLC) with UV [7] or with mass spectrometric detection [8]. Another method assesses the rate of MAGL-catalyzed hydrolysis of *p*-nitrophenyl alkyl esters by monitoring the liberation of *p*-nitrophenol [9]. Furthermore, a fluorescence-based assay has been developed applying 7-hydroxycoumarinyl arachidonate (7-HCA) [10], as well as an HPLC method with fluorescence detection, using as fluorogenic probe 1,3-dihydroxypropan-2-yl-4-pyren-1-ylbutanoate [11]. We have previously reported an efficient protocol for a MAGL continuous assay, based on a new long-wavelength fluorogenic substrate, 7-hydroxyresorufinyl arachidonate **1g** [12]. Here we report the synthesis and the evaluation, as MAGL assay substrates, of 10 alkyl esters of fluorescent resorufin with the aim to propose a high-throughput screening (HTS) method for MAGL activity based on a new red fluorogenic probe. Essential to the success of any HTS program that seeks to discover modulators of the function of specific proteins, is the development of a high-quality screening, which requires an accurate, and homogeneous biochemical readout of protein activity, robust assay reproducibility and appropriate sensitivity [13,14]. Furthermore, possible interferences due to the concentrations of library compounds used as protein activity modulators in the assay could occur, so shifting the emission wavelength to the red reduces these interferences [15]. It must also be underlined that a suitable substrate has to be readily accessible, stable in solution and with low rate of spontaneous hydrolysis.

**Figure 1:**
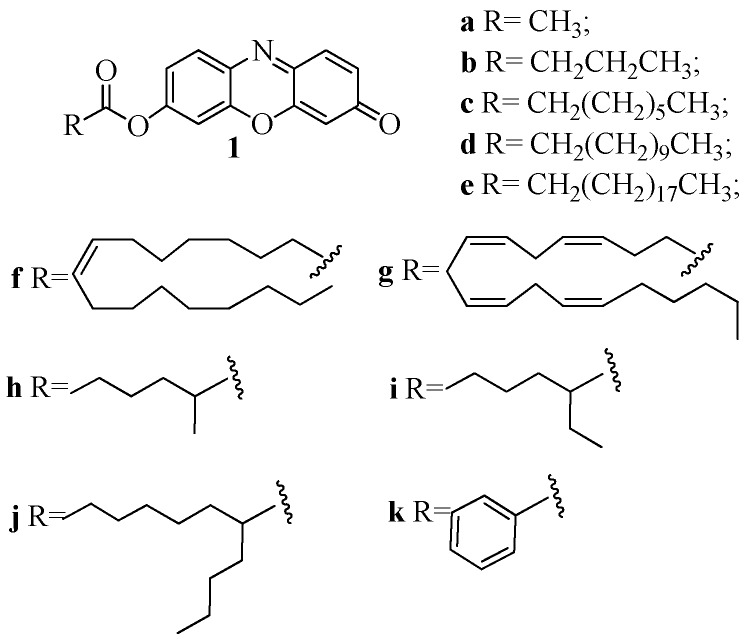
Structures of 7-hydroxyresorufynil esters: acetate (**1a**), butyrate (**1b**), octanoate (**1c**), dodecanoate (**1d**), icosanoate (**1e**), oleate (**1f**), arachidonate (**1g**), 2-methylhexanoate (**1h**), 2-ethylhexanoate (**1i**) 2-butyloctanoate (**1j**), benzoate (**1k**).

We have previously reported an efficient protocol for a MAGL continuous assay, based on a new long-wavelength fluorogenic substrate, 7-hydroxyresorufinyl arachidonate **1g** [12]. Here we report new esters of fluorescent resorufin with different classes of acyl chains such as linear, i.e., acetate (**1a**), butyrate (**1b**), octanoate (**1c**), dodecanoate (**1d**), icosanoate (**1e**), oleate (**1f**), branched, i.e., 2-methylhexanoate (**1h**), 2-ethylhexanoate (**1i**) and 2-butyloctanoate (**1j**), and aromatic, i.e., benzoate (**1k**) (Figure 1) and compared them with **1g**. Moreover, we investigated the MAGL substrate specificity depending on the acyl chain, and determined the kinetic constants. The differences of substrate interaction with MAGL active site were also analyzed using structural in-silico techniques.

## 2. Results

### 2.1. Synthesis of ***1a–k***

For the synthesis of fluorogenic compounds, two different methods have been followed. While the synthesis of the esters **1a–g** involves passing through the formation of the acyl chloride, the compounds **1h–k** required Steglich esterification [16]. In the case of fatty acid with branched acyl chain such as 2-methylhexanoic acid, 2-ethylhexanoic acid and 2-butyloctanoic acid the symmetrical anhydrides were isolated instead of the esters. Therefore, the use of oxalyl chloride has to be avoided with sterically hindered carboxylic acids. In the Steglich esterification indeed, the addition of 3 mol % DMAP accelerates the DCC-activated esterification of carboxylic acids with alcohols to such an extent that formation of side products is suppressed. Synthesized molecules were completely characterized by 1D NMR, as well as 2D NMR homocorrelation (COSY) and heterocorrelation (HMQC and HMBC). The purity of all compounds measured with NMR was greater than 98%.

### 2.2. Substrates Screening

**Figure 2:**
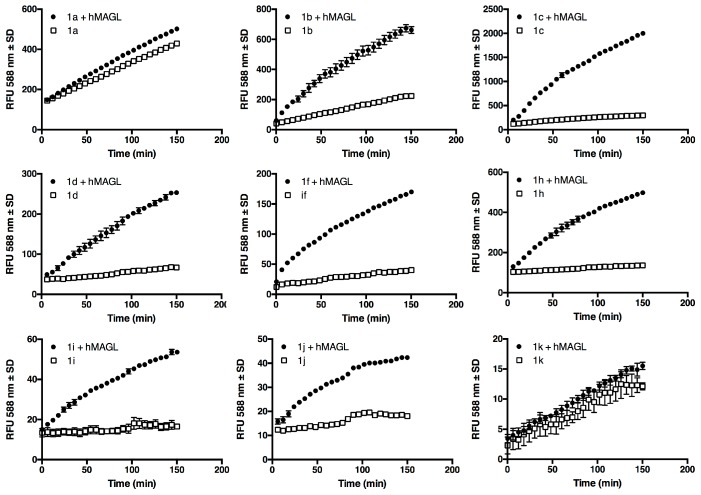
Time course of **1a**–**d**, **1f**, and **1h**–**k** hydrolysis by human recombinant monoacylglycerol lipase *(h*MAGL). 25ng/well of *h*MAGL (circles) or buffer alone (empty squares) were incubated in 96-well black plate, at room temperature, with 5 μM of each compound in total volume of 100 μL/well, as described in the experimental section. Fluorescence was measured at indicated time points with a Jasco FP-8300 fluorometer (Jasco Europe, Cremella (LC) Italy) using the kinetic mode (λ_ex_ = 571 nm, λ_em_ = 588 nm, slit = 5.0). Data are mean ± standard error of independent experiments.

Screening experiment were performed at 5 μM in Tris-HCl containing EDTA (10% DMSO) in the presence of 25 ng of human recombinant MAGL (*h*MAGL). Typical MAGL assays use bovine serum albumin (BSA) since it assists in the solubility of the substrate and might prevent its non-specific binding to the plastic labware [10]. We decided not to use BSA because it is not compatible with lipase assay due to its well-known esterase-like activity [17]. As reported in Figure 2, at 5 μM compound **1a** shows a considerable spontaneous hydrolysis, compound **1b** a smaller one while all the other esters seem to be stable. During the set-up of the assay conditions also spontaneous hydrolysis of **1a**–**d**, and **1h**–**k** at all concentration used for kinetic experiments (data not shown) have been evaluated. At 25 μM, the highest concentration assessed (Supplementary Figure S1), compounds **1a**–**c** show a hydrolysis rate increment proportional to the concentration as expected, even if almost negligible for **1c**. Compounds **1i**–**k** seems to be still stable with no significant increment in hydrolysis rate, while compounds **1d**–**h** show an atypical behavior, indeed, to a five-fold increase of the ester concentration correspond a less increment in the rate of hydrolysis. For this reason, the solubility in aqueous buffer of substrates **1d**–**h** at 25 μM was verified by dynamic light scattering (DLS) analyses (Supplementary Figure S3). DLS analyses revealed the presence of micelles and/or aggregates that are formed at this concentration, due to the hydrophobic nature of the substrates.

### 2.3. Kinetic Studies

**Figure 3:**
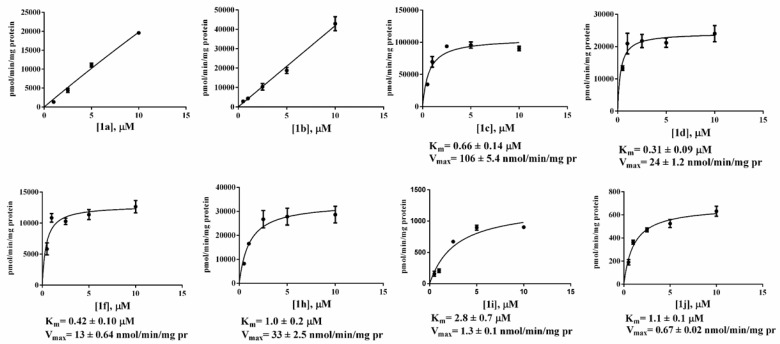
Action of *h*MAGL on different concentrations of **1a**–**d**, **1f**, **1h**–**j**, in the presence of DMSO (10%, *v*/*v*). The reactions were conducted in 50 mM Tris-HCl buffer (pH 7.4, 1 mM EDTA) with 25 ng/well of *h*MAGL. Values are the means of triplicates ± standard error. The kinetic parameters *K*_m_ and *V*_max_ were determined via computer-assisted nonlinear regression analysis using GraphPad Prism 6.0c (GraphPad Software, San Diego, CA 92108).

The amount of enzyme used in the assay was optimized to 25 ng/well to maintain linearity over time and to maintain substrate consumption below 10% to adhere to the assumptions of the Michaelis–Menten equation [12]. Enzyme kinetics were analyzed by measuring product formation rates in the presence of different amounts of the substrates. Standard curves, showing fluorescence response vs. fluorophore concentration, were constructed from a resorufin dilution series, transforming relative fluorescence units (RFU) into picomoles of resorufin. Figure 3 reports the action of *h*MAGL on different concentrations of all synthesized substrates. To evaluate the real enzymatic activity, the amount of free resorufin released from the spontaneous hydrolysis of the substrate was subtracted.

The initial linear part of the resulting curve and its slope were considered to measure the enzyme activity [18]. Michaelis–Menten kinetic parameters, K_m_ and V_max_ values, were derived with Prism GraphPad software, Version 6.0c applying a nonlinear regression analysis and Michaelis-Menten fit (Table 1). As shown in Figure 3, fluorometric determination of the kinetic constants for compounds **1a** and **1b** was impossible. For all other compounds, Michaelis-Menten curves were constructed considering *h*MAGL hydrolysis at five concentrations (0.5, 1.0, 2.5, 5.0, 10.0 μM) and the obtained results for K_m_ and V_max_ are reported in Table 1. Among new compounds, **1j** has the best fit with Michaelis-Menten regression but has a very low rate of hydrolysis.

Kinetic parameters reported in Table 1 highlight the substrate preference of *h*MAGL. Of all the compounds newly synthesized, the fastest hydrolyzed was **1c**. Compound **1e** has a very low solubility in DMSO (<0.5 nM) and could not be assessed as *h*MAGL substrate. Actually, even LogD value (Table 1) highlights higher affinity of this compound to non-polar organic solvents. Substrate **1k** was excluded from further investigations due to its inappreciable hydrolysis by *h*MAGL. All substrates have K_m_ values ranging from 0.31 to 2.8 μM, in accordance to that of 0.87 μM already calculated for **1g** [12]. Maximum velocities are similar as well, ranging from 0.67 to 33 nmol/min/mg protein, with the exception of **1j** that displays a very low hydrolysis rate with a V_max_ of 0.67 nmol/min/mg protein. Interestingly, **1c** has a V_max_ of 106 nmol/min/mg protein. For this reason, compound **1c** was selected as fluorogenic substrate for the set-up of a new method.

### 2.4. Validation of ***1c*** for Screening Assay

**Figure 4:**
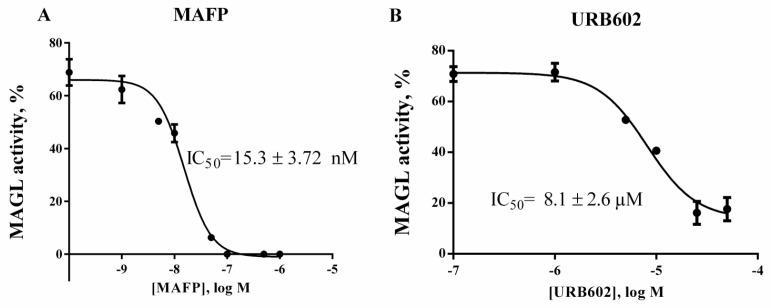
Inhibition of human recombinant MAGL with known inhibitors: methyl arachidonyl fluorophosphonate (**A**), URB602 (**B**). The assay was carried out as described in Experimental procedures section. Data derived from two independent experiments performed in triplicate and calculated as non-linear regressions using sigmoid dose-response setting with variable Hill slope by GraphPad Prism 6.0c. In panel B 100% inhibition is not achieved due to the low solubility of URB 602 in assay conditions.

In order to validate compound **1c** for HTS two known MAGL inhibitors, URB602 and Methyl arachidonylfluorophosphonate (MAFP) [7,19] were used. The dose-response curves are shown in Figure 4. After incubation of *h*MAGL with the inhibitor, **1c** was rapidly added and fluorescence read for 30 min. The activity of *h*MAGL was calculated as described for the kinetic assay. IC_50_ for URB602 was found to be 8.1 μM, which is in line with literature data [20]. As already known, MAFP had the lowest IC_50_ value, our experiments gave an IC_50_ of 15.3 nM, which is in the range of values indicated in literature [12,20].

### 2.5. Docking Studies

**Figure 5:**
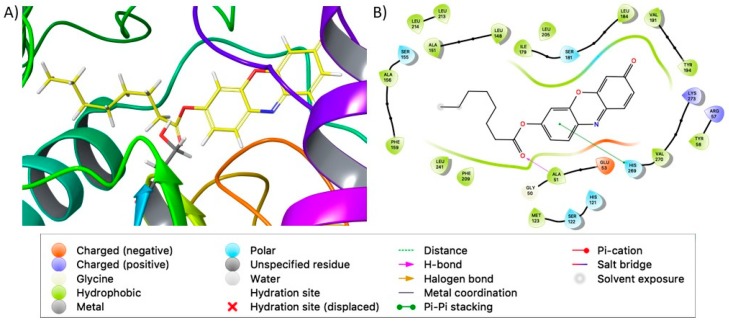
(**A**) Docking poses of compound **1c** into the MAGL binding site. MAGL is shown in ribbon representation; tested compound and Ser122 are shown in stick representation and hydrogens are hidden. (**B**) Ligand interaction diagram of compound **1c** into the MAGL binding site.

The identified binding site corresponds to the catalytic active site identified by Lauria et al. [21] Table 1 reports the Glide XP docking score and MM-GBSA binding energies for the top scoring pose of each tested compound, showing that all of them are able to bind MAGL in its catalytic site. Docking simulations provided overlapping poses for all the tested compounds and a good superposition of their common resorufin group, confirming the accuracy of this approach. Moreover, the carbonyl group for all the tested compounds, except for **1j**, overlaps the carbonyl group of the natural substrate 2-AG in proximity to Ser122, according to Lauria et al. [21]. In particular, Ala51, Ser122 and Met123 establish hydrogen bond interactions with the carbonyl and ester oxygen atoms (Figure 5), while His121 and His269 are involved in π–π interaction with the resorufin group, as reported in Table S2 of Supplementary. As shown in Figure 5, the alkyl chains of the resorufine ester accommodate in a well-shaped pocket surrounded by hydrophobic/aromatic residues, such as Leu148, Leu213, Leu214, Ile179, Ala151, Ala156, Phe159, Phe209. Accordingly, in silico results also underline the importance of the contribution of the hydrophobic interactions in the stability of the complexes.

## 3. Discussion

Here we present 7-hydroxyresorufinyl octanoate (**1c**) as a new fluorogenic substrate to evaluate MAGL activity by HTS. Although numerous assay methodologies have been published in the past decades for measurement of MAGL activity, few of them allow the screening of a large number of compounds showing high sensibility, high throughput, and low assay cost. In order to overcome these issues, we investigated the use of an alternative substrate for measuring MAGL activity. The native substrates for MAGL include 2-AG (C20:4) and 2-oleoyl glycerol (2-OG, C18:1) both containing long hydrocarbon fatty acid chains with relatively unstable cis olefins. Nevertheless, MAGL is able to hydrolyze a vast array of esters structurally very different, such as *p-*nitrophenyl esters [9] or 7-hydroxycoumarinyl esters [22]. We chose resorufine as fluorescent probe because its derivatives have proven to be an excellent fluorescence marker and have the advantages to be hardly susceptible to background signals. Often pharmacological and biochemical research need to measure enzymatic activity in crude cell lysate or turbid solution [23], the shift of emission wavelengths at higher values give less interference [24]. In the current approach, 10 fluorogenic alkyl esters of resorufin with different acyl chains are synthesized and evaluated as MAGL assay substrates (Figure 1). Selected compound **1c** ensure lower background signals, thanks to resorufine in comparison with 7-methylumbelliferone, higher stability and lower costs if compared to arachidonoyl or oleyl derivatives.

**Figure 6:**
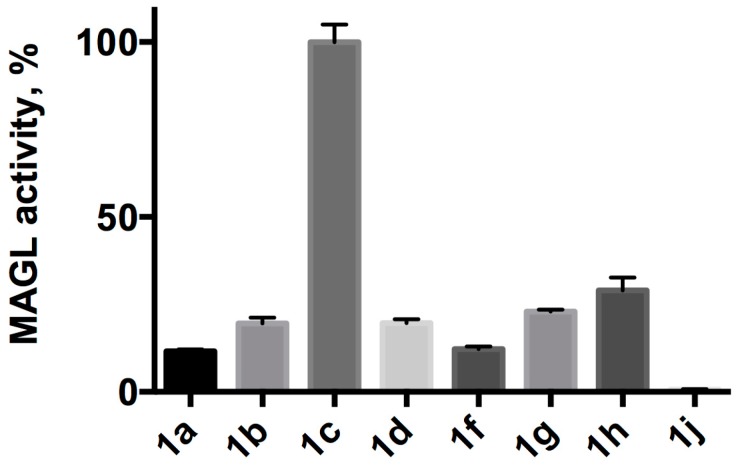
Normalized hydrolysis of different substrates by *h*MAGL. Release of 7-hydroxyresorufin upon hydrolysis of different resorufynil esters (each at a concentration of 5 μM) by 25 ng *h*MAGL was measured in triplicates, the slopes of the regression lines were averaged and then normalized against the slope of the most efficiently hydrolyzed compound. The error bars represent the standard deviation of the results. The following compounds were used as drawn below the bars: acetate (**1a**), butyrate (**1b**), octanoate (**1c**), dodecanoate (**1d**), oleate (**1f**), arachidonate (**1g**), 2-methylhexanoate (**1h**), and 2-butyloctanoate (**1j**). The specific activity of the *h*MAGL preparation was 241.9 U/mg.

MAGL fluorescence assays were carried out in the presence of 5 μM substrate, 10 % DMSO and at pH 7.4, a value that attempts to mimic the native intracellular conditions. Diverging too far from the physiological pH could affect the interactions between the enzyme and the substrates, thus giving an altered view of the potential biochemical and cellular activity of these compounds. Moreover, this pH value can be maintained by various buffers, such as Tris-HCl, PBS, sodium phosphate, Mops and Hepes, and allowed us a prompt comparison with previous data [12]. Among the synthesized compounds, linear acylic chains of up to 12 carbon atoms are substrates for MAGL, as well as the derivative of oleic acid, as expected, since 2-OG, an oleic acid derivative, is one of the native substrates of the protein. The addition of just two more carbons to the side chain makes the hydrolysis of this compound almost non-existent. The same result is obtained when the branching becomes as long as four carbon atoms. When in aqueous buffer, **1a** and **1b** show the highest rate of spontaneous hydrolysis among our compounds (Figure 2), to the point that fluorometric determination of their kinetic constants proved impossible (Figure 3). For resorufin acetate **1a**, this behavior was already reported by Maeda and colleagues [25], leading to the impossibility of using that compound as a probe. Even if **1b** showed an inferior hydrolysis rate than **1a** (Figure 1), this compound is unsuitable for the achievement of Michaelis-Menten conditions in an aqueous environment (Figure 3) [26]. On the other hand, compounds with a long linear chain or with a branched side chain, showed low to negligible hydrolysis. These differences may be explained with the susceptibility of acyl resorufins towards nucleophilic attack [27]. Short linear side chains (**1a**, **1b**) leave the α carbon exposed to the attack of water, while side chains with branches next to the carboxyl group (**1h**, **1i**, **1j**) exert a steric hindrance on water molecules, protecting the ester group from hydrolysis. A similar phenomenon reasonably happens with medium (**1c**, **1d**) and long (**1f**, **1g**) alkyl chains. When in an aqueous environment, the alkyl chain folds on itself via van der Waals interactions next to the carboxyl group, thus mimicking the side branch protecting effect. Moreover, the formation of aggregates, as highlighted by DLS analyses (Supplementary Figure S2), could be a reasonable explanation for the unusual behavior observed in spontaneous hydrolysis experiments of compounds **1d**–**h** where to a five-fold increase of ester concentration corresponds a less increase in the rate of the hydrolysis. In Figure 6, hydrolysis of different substrates by MAGL, normalized against compound **1c**, is reported to better underline the high difference between the hydrolysis rate displayed by **1c** and the other proposed MAGL substrates.

The presence of aggregates in the substrates’ solutions at 25 μM concentration prompted us to consider only solutions until 10 μM concentration for calculations of kinetic parameters. Observing obtained results (Figure 3), **1f** gives a good fit with Michaelis-Menten type kinetics indeed, its side chain, the same of natural substrate 2-OG, seems to allow a better accommodation in the enzyme active site. It is possible that the enhanced rigidity of the alkyl chain, conferred by the presence of the double bond, guides the substrate to a conformation favorable to enzymatic hydrolysis, hindering disadvantageous folding in the enzyme active site.

The scale of Y axis (Figure 3), and the V_max_ and K_m_ values (Table 1), highlight how steric hindrance, due to the different length of acyl side chain on C2, reduce the rate of substrate hydrolysis. Interestingly, **1j** shows a Michaelis-Menten kinetic behavior even if with very low hydrolysis rate. In this case, the bulkier side chain likely exerts an even stronger effect of steric hindrance, which does not allow reaching the concentration needed to manifest the inhibitory effect. Compound **1c** proved to be a very interesting substrate for MAGL. Indeed, with respect to the previously described arachidonate **1g** [12] and oleate **1f,** the two resorufine derivatives of the MAGL natural substrates, **1c** shows slightly higher XP Glide docking score, MM-GBSA binding energy and K_m_ values. In the molecular docking poses, **1c** resorufin perfectly overlaps the **1g** one, while the linear side chain of **1c** mimics the 2-AG side chain. Focussing our attention specifically on the difference in activity pointed out for compound **1c** versus **1b,** as already reported in the Results section and in Figure S4, a significant relative difference in the extension of the hydrophobic interactions can be one of the factors contributing to the different stability of the complexes, as shown by the different of the solvation/desolvation energy reported in the Prime MM-GBSA energy column in Table 1. The V_max_ for **1c**, however, is much higher than that of **1g**, as indicated also by the direct comparison of the catalytic speed of the two substrates. In addition, the smaller substrate **1c** has superior aqueous solubility properties and contains no potential labile cis olefins. Therefore, the signal to background ratio of **1c** (seven to one) is suitable for the screening for MAGL inhibitors in high-throughput screening. For all these reasons, **1c** was selected and validated as new red fluorogenic probe for the HTS method.

MAFP and URB602, two well-known serine hydrolase and MAGL inhibitors, were chosen for validation [7,19]. After a 60 min pre-incubation of the enzyme with the inhibitor, the IC_50_ values for both MAGL inhibitors using *h*MAGL and **1c** (5 μM) were obtained and compared with those reported in literature [12,20]. In order to evaluate and validate the assay for its potential in high-throughput screening applications, we further determined the Z-factor [28], a dimensionless statistical parameter that reflects both the assay signal dynamic range and the data variation associated with the signal measurements. The assay was highly reproducible with an overall average Z′ value of 0.86. The objective of this study was to identify a substrate readily accessible, stable in solution and with low rate of spontaneous hydrolysis for realizing an HTS method for MAGL activity using human recombinant MAGL. It should be considered that MAGL belongs to the group of serine hydrolases (SHs), a superfamily of enzymes able to cleavage ester, amide, or thioester bonds of protein, peptide, and small molecule substrates [29] including esterases, lipases, peptidases, and amidases [30]. Within this family, there are the SHs that regulate the biosynthesis and degradation of two major endocannabinoids, AEA and 2-AG, as well as other lipid-metabolizing serine hydrolases that are ubiquitously distributed [31]. Therefore, in the potentiality to apply **1c** as a probe for experiments performed in complex biological systems, as living cells or cell lysates, the hydrolysis of the probe **1c** by other SHs enzymes should be considered. These results demonstrated that our fluorometric method could be successfully applied to identify compounds that modulate MAGL activity.

## 4. Materials and Methods

### 4.1. Chemicals

Arachidonic acid, resorufin, and all other reagents and solvents were purchased from Sigma-Aldrich. Reactions progress was monitored by analytical thin-layer chromatography (TLC) on pre-coated aluminum foils. Monoacylglycerol lipase (human recombinant, 50 μg) was purchased from Cayman Chemical. All steps, which included resorufin, were carried out protecting the compound from light.

Fluorescence signals were recorded by a Jasco FP-8300 fluorometer using the kinetic mode (λ_ex_ = 571 nm, λ_em_ = 588 nm, slit = 2.5 nm in both cases) in black, flat-bottomed, 96-well polystyrene microtiter plates.

^1^H-NMR spectra were recorded in CDCl_3_ (isotopic enrichment 99.95%) solutions at 300 K using a Bruker AVANCE 500 instrument (Bruker Italia Srl, Milan, Italy) (500.13 MHz for ^1^H, 125.76 MHz for ^13^C) using 5 mm inverse detection broadband probes and deuterium lock. Chemical shifts (δ) are given as parts per million relative to the residual solvent peak (7.26 ppm for 1H) and coupling constants (J) are in Hertz (Supplementary Table S4).

### 4.2. Synthesis

#### 4.2.1. Synthesis of 7-Hydroxyresorufinyl-Derivatives **1a**–**1g**

To a solution of the opportune carboxylic acid (0.46 mmol) in dry CH_2_Cl_2_ (2 mL), oxalyl chloride (58 μL, 0.69 mmol) in dry CH_2_Cl_2_ (0.5 mL) was added dropwise at 0 °C under stirring. *N,N*-dimethylformamide (DMF, 1 drop) was added next. The reaction mixture was stirred at room temperature for 3 h and then concentrated under vacuum, giving crude acyl chloride. This residue was dissolved in dry CH_2_Cl_2_ (1 mL) and added dropwise to an ice-cold suspension of resorufin (50 mg, 0.23 mmol) and triethylamine (48 μL, 0.35 mmol) in dry CH_2_Cl_2_ (3 mL) and then stirred overnight at room temperature. After dilution with CH_2_Cl_2_ the salts residues were removed by filtration obtaining a brick-red solution; that was washed with 0.5 M HCl (2 mL) and saturated NaHCO_3_ (2.5 mL), dried on anhydrous Na_2_SO_4_ and concentrated to give the crude product that was purified with column chromatography on silica gel (Supplementary Table S1). Yields 62–92%.

#### 4.2.2. Synthesis of Resorufin Esters **1h**–**1k**

To a stirred solution of the opportune carboxylic acid (0.46 mmol) in dry CH_2_Cl_2_ (3 mL), were added DMAP (2.5 mg. 0.02 mmoli) and resorufin (50 mg, 0.23 mmol). DCC (72 mg, 0.35 mmoli) was added to the reaction mixture at 0 °C. Stirring was continued for 5 min at 0 °C and for 3 h at 20 °C. Precipitated urea was then filtered off and the filtrate evaporated down in vacuo. The residue was taken up in CH_2_Cl_2_ and, if necessary, filtered free of any further precipitated urea. The CH_2_Cl_2_ solution was washed twice with 0.5 M HCl (2 mL) and saturated NaHCO_3_ (2.5 mL), dried on anhydrous Na_2_SO_4_ and concentrated to give the crude product that was purified by column chromatography on silica gel, yields 82–92% (Supplementary Table S1).

### 4.3. Stability of the Substrates

The stability of all substrates was tested in 50 mM Tris-HCl buffer (pH 7.4, 1 mM EDTA), as previously reported [12]. Briefly, 10 μL of the substrates dissolved in DMSO (final concentration 5 μM) were added to 90 μL of the buffer. The fluorescence increase at 588 nm was monitored at intervals of 1 min over 90 min at rt., using a Jasco FP-8300 fluorometer (λ_ex_ = 571 nm, λ_em_ = 588 nm).

### 4.4. Substrates Screening

*h*MAGL activity was monitored following the increase of resorufin fluorescence (λ_ex_ = 571 nm, λ_em_ = 588 nm), at intervals of 1 min. *h*MAGL (specific activity 241.9 U/mg) was diluted to 250 ng/mL in 50 mM Tris-HCl buffer (pH 7.4), with 1mM EDTA (reaction buffer). Each reaction well contained 80 μL Tris-HCl 50 mM with EDTA 1 mM, 10 μL of reaction buffer with 25 ng *h*MAGL and the putative substrate dissolved in 10 μL of DMSO (final concentrations 5 μM or 25 μM for all other substrates), total volume 100 μL [12]. In addition, resorufin calibration curve and negative control, containing 90 μL of reaction buffer and no *h*MAGL, were measured. All resorufin ester solutions were freshly prepared in DMSO.

### 4.5. Kinetic Assays of hMAGL

Reagent solutions for each substrate were prepared diluting first 5 mM stock solution with DMSO (5 μM, 1 μM, 25 μM, 50 μM, 100 μM and 250 μM), and then 1:9 in the reaction buffer. For kinetic experiments 10 μL of the reaction buffer, containing 25 ng of *h*MAGL, and 90 μL of the appropriate reagent solution were added to each well. The final concentrations were for *h*MAGL 25 ng/100 μL (7.6 nM), for all substrates 0.5 μM, 1 μM, 2.5 μM, 5 μM, 10 μM, and 25 μM, and DMSO 10%. For negative controls 10 μL of reaction buffer were added instead of enzyme. In addition, blank samples (90 μL of reaction buffer, 10 μL DMSO) were analyzed. Fluorescence was recorded at room temperature for 50 cycles with a cycle time of 3 min. A standard curve was generated by plotting fluorescence of five concentrations of resorufin (0.02 μM, 0.1 μM, 0.5 μM, 1 μM, 5 μM) prepared by diluting DMSO stocks in reaction buffer (10% DMSO).

### 4.6. Data Analysis

All experiments were performed in triplicate and independently replicated at least once. The curve generated from the hydrolysis of the substrates was used to convert raw fluorescence data into nmol/mL/min of resorufin produced. The values of negative controls were subtracted from the enzymatic curve. The initial velocities were determined from the linear portion of the resulting curve. Kinetic data were elaborated using GraphPad Prism 6.0c and Microsoft Excel graphing software; kinetic parameters K_m_ and V_max_ were calculated using GraphPad, applying a nonlinear regression analysis (Michaelis-Menten). The quantitative data were calculated as means ± standard errors. The Z′-factor was calculated using the equation Z′ = 1 − (3σh + 3σl)/|μh − μl|, where σh and σl are the standard deviations of the high and low signal controls, respectively, and μh and μL are the mean signal intensities of the high and low signal controls, respectively [28].

### 4.7. Validation of ***1c*** for Screening Assay

MAFP and URB602 were chosen for the method validation due to their well-known MAGL inhibitory activity [7,19]. To prepare inhibitors stock solutions, commercial MAFP solution (10 mg/mL in ethanol) was diluted to 200 μM in DMSO and 15 μM URB602 solution in DMSO was obtained from powder. Eight different working solution were then prepared by dilution with DMSO. 10 μL of diluted *h*MAGL solution containing 25 ng of the enzyme and 10 μL of the appropriate MAFP or URB602 solution were added to wells of a 96-well plate and the volume was adjusted to 95 μL with reaction buffer (Tris-HCl 50 mM with EDTA 1 mM). In control wells, 10 μL of DMSO were added instead of inhibitor solution and the black samples containing only reaction buffer and DMSO (10%) also were prepared. Final concentrations of MAFP were 1.0 μM, 500.0 nM, 100.0 nM, 50.0 nM, 10.0 nM, 5.0 nM, and 0.1 nM; final concentrations of URB602 were 75.0 μM, 50.0 μM, 25.0 μM, 10.0 μM, 5.0 μM, 1.0 μM, and 100.0 nM. A 100.0 μM **1c** working solution was prepared by diluting a 5.0 mM DMSO stock 1:50 in DMSO. After 60 min of incubation at 25 °C, 5.0 μL of **1c** working solution was added to each well to give a final substrate concentration of 5.0 μM (10% DMSO). Fluorescence was recorded at room temperature for 30 cycles, with a cycle time of 1 min. All experiments were performed in triplicate and independently replicated at least once and the mean of the three obtained values was used for calculation. The mean fluorescence value of a blank was subtracted from the value of each sample and control well to normalize data at each time point, the mean value of control wells was subtracted to the mean value of each sample. From the slop of kinetic curves, residual enzymatic activity was calculated and IC50 values were obtained by non-linear regression analysis of log[concentration]/inhibition curves. IC50 was determined as the concentration of inhibitor that results in an initial velocity 50% that of the sample containing no inhibitor. IC50 was used along with previously calculated K_m_ to determine K_i_.

### 4.8. In Silico Molecular Docking Simulations

All the computational procedures were carried out by the Schrödinger Small-Molecule Drug Discovery Suite 2018-01 (Schrödinger, Cambridge, USA). The crystallographic structure of the catalytic domain of *h*MAGL was downloaded from the RCSB PDB (PDB ID: 3PE6, resolution of 1.35 Å) [32]. Since the selected MAGL crystallographic structure presents three engineered mutations for increasing the quality of the diffracting crystal, the Schrödinger Protein Refinement tool was used to mutate Ala36, Ser169 and Ser176 in Lys36, Leu169 and Leu176, respectively. The wild-type MAGL structure was then energy minimized using the Schrödinger Protein Preparation Wizard in order to fix structural issues in the three-dimensional (3D) structure. Tested ligands were built through the Schrödinger Maestro Build Toolbar and prepared for docking by the Schrödinger Ligand Preparation, generating the stereoisomers of **1h**, **1i**, and **1j**. A receptor grid, which defines the MAGL active site, was generated via the Schrödinger Receptor Grid Generation, centring a cubic box, with an edge of 20 Å, on the co-crystallized inhibitor. The molecular docking procedure was carried out by the Schrödinger Glide Docking in the “extra precision (XP)” mode in order to evaluate the ability of the tested ligand to bind the MAGL catalytic domain, keeping only the 20 top-scoring poses. The top-scoring solution for each ligand was submitted to the Schrödinger Prime MM-GBSA, which integrates molecular mechanics energies combined with the generalized Born and surface area continuum solvation [33] in order to compute ligand binding and ligand strain energies for a set of ligands and a single receptor.

### 4.9. Dynamic Light Scattering Analyses

Dynamic light scattering experiments were performed in a custom modified setup (Scitech 100). The wavelength of the excitation light is λ = 532 nm and the scattered light was collected at a constant angle θ = 90°. Both the excitation light and the scattered light were introduced and fetched by means of polarization-maintaining fibers. The sample was put in a cylindrical glass capillary (O.D. = 3 mm I.D. 2.4 mm) and sealed with a silicon cap. The intensity time autocorrelation was obtained using g2 (τ) a digital correlator (flex-03d Correlator.com). Each g2 (τ) was obtained after averaging 600 s and for each experiment three g2 (τ) were acquired.

## 5. Conclusions

We present here the set-up and validation of a new HTS method for MAGL activity based on a red fluorogenic probe. Starting from the synthesis of a few long-wavelength fluorogenic compounds, characterized by different acylic side chains, and their application to the fluorometric determination of MAGL activity, 7-hydroxyresorufinyl octanoate (**1c**) was selected as the best substrate for the HTS method. **1c** is the eligible substrate among the compound tested, being the substrate with the higher rate of hydrolysis and the best K_m_ and V_max_ values. in-silico docking studies show the favorable interactions between **1c** and MAGL active site. Moreover, compound **1c** can be easily prepared in milligram and gram scale and is very stable in solution as demonstrated by the low rate of spontaneous hydrolysis. The probe **1c** was validated using the well-known MAGL inhibitors URB602 and MAFP, in assay condition.

In conclusion, the fast, sensitive, and accurate method described in this study is a powerful new tool for the high-throughput assay of MAGL activity and screening of MAGL modulators.

## Figures and Tables

**Table 1:** List of tested substrates with kinetic and modeling parameters.

Compound	LogD at pH 7.4 ^a^	K_m_ (μM)	V_max_ (nmol/min/mg protein)	Docking Score	MM-GBSA	Fitting Model	r^2^
**1a**	1.69	n/a	n/a	−6.7	−52.4	n/a	n/a
**1b**	2.83	n/a	n/a	−7.7	−60.5	n/a	n/a
**1c**	4.61	0.66 ± 0.14	106 ± 5.4	−8.2	−72.0	M-M	0.8488
**1d**	6.39	0.31 ± 0.09	24 ± 1.2	−10.5	−81.4	M-M	0.6731
**1e**	9.94	n/a	n/a	−9.8	−67.8	n/a	n/a
**1f**	8.69	0.42 ± 0.1	13 ± 0.64	−10.8	−73.2	M-M	0.7391
**1g**	8.50	0.87 ± 0.13	25.8 ± 0.88	−10.7	−76.0	M-M	0.9908
**1h**	4.26	1.09 ± 0.02	33 ± 2.509	−8.5	−60.3	M-M	0.8859
**1i**	4.71	2.8 ± 0.7	1.3 ± 0.1	−8.8	−62.8	M-M	0.9236
**1j**	6.49	1.1 ± 0.1	0.67 ± 0.03	−8.4	−65.4	M-M	0.9450
**1k**	3.74	n/a	n/a	−8.9	−63.2	n/a	n/a

**^a^** Data generated using the Chemicalize https://chemicalize.com/ (developed by ChemAxon, http://www.chemaxon.com).

## Supplementary Materials

The following are available online. Table S1. Mobile phases used for the separation of synthesis products. Table S2. Ligand: MAGL interactions. Table S3. Decay times and corresponding radius. Table S4. Assignment of ^1^H and ^13^C-NMR Chemical Shift in CDCl_3_. Figure S1. Comparison between spontaneous hydrolysis of **1a**–**d**, **1f**, and **1h**–**k**. Figure S2: Time correlation functions for the scattered intensity (at θ = 90°). Figure S3. Binding poses of tested compounds into the MAGL binding site. ^1^H- and ^13^C-NMR spectra of compounds **1a**–**k**. Figure S4. MAGL complexes with compounds **1b** and **1c.**
