# Supplementary material for: Set-Up and Validation of a High Throughput Screening Method for Human Monoacylglycerol Lipase (MAGL) Based on a New Red Fluorescent Probe
Source: Molecules. 2019 Jun 15;24(12):2241. doi: 10.3390/molecules24122241 (PMC6631453; doi:10.3390/molecules24122241)
Supplement: Supplementary file 1 [file molecules-24-02241-s001.pdf]

Supplemental Material

"Set-up and Validation of a High Throughput Screening Method for Human Monoacylglycerol Lipase (MAGL) Based on a New Red Fluorescent Probe".

Matteo Miceli[1], Silvana Casati [1], Roberta Ottria [1], Simone Di Leo [2], Ivano Eberini [3], Luca Palazzolo [3], Chiara Parravicini [3], and Pierangela Ciuffreda [1,*]

[1] Dipartimento di Scienze Biomediche e Cliniche "Luigi Sacco", Università degli Studi di Milano, Via G.B. Grassi 74, 20157 Milano, Italy; matteo.miceli@unimi.it (M.M.), silvana.casati@unimi.it (S.C.), roberta.ottria@unimi.it (R.O.); pierangela.ciuffreda@unimi.it (P.C.);

[2] Dipartimento di Biotecnologie Mediche e Medicina Traslazionale, Università degli Studi di Milano, Via Fratelli Cervi 93, 20090, Segrate (MI), Italy; simone.dileo@unimi.it (S.DL.);

[3] Dipartimento di Scienze Farmacologiche e Biomolecolari, Via Balzaretti 9, 20133 Milano, Università degli Studi di Milano, Italy; ivano.eberini@unimi.it (I.E.), luca.palazzolo@unimi.it (L.P.), chiara.parravacini@unimi.it (C.P.);

Table of Contents

Tables

Table S1. Mobile phases used for the separation of synthesis products. Pag. 2
Table S2. Ligand: MAGL interactions. Pag. 3
Table S3. Decay times and corresponding radius. Pag. 4
Table S4. Assignment of ^{1}H and ^{13}C-NMR Chemical Shift in $CDCl_3$. Pag. 7-10

Figures

Figure S1. Comparison between spontaneous hydrolysis of **1a-d**, **1f**, and **1h-k** at lowest (circles) and highest (squares) concentrations assessed (5 μM and 25 μM). For all compounds, data have been normalized subtracting the t0 fluorescence value from all the next fluorescence value read. Data are mean ± standard error of independent experiments. Pag. 3
Figure S2: Time correlation functions for the scattered intensity (at θ = 90°). Red dashed lines: double exponential fit. The fastest decay time is of the order of fractions of μs which does not correspond to diffusive motion. Whereas the slower decay times reveal nano-sized particles. The corresponding decay times and radius are reported in Tab.3S. Pag. 4
Figure S3. Binding poses of tested compounds into the MAGL binding site. MAGL is represented as ribbons; tested compounds and Ser122 are represented as sticks without hydrogen atoms. Pag. 5
Figure S4: MAGL complexes. Compound **1b** (A) and **1c** (B) are shown in orange and yellow sticks, respectively; only MAGL residues participating to the binding site are reported in stick representation; green traces point out residues involved in hydrophobic interactions. Pag. 5
Figure S5. IUPAC numbering of 7-hydroxyresorufinyl moiety. Pag. 6

^{1}H and ^{13}C-NMR data

^{1}H and ^{13}C NMR spectra of 7-hydroxyresorufinyl-acetate (**1a**) Pag. 11
^{1}H and ^{13}C NMR spectra of 7-hydroxyresorufinyl-butyrate (**1b**) Pag. 13
^{1}H and ^{13}C NMR spectra of 7-hydroxyresorufinyl-octanoate (**1c**) Pag. 15
^{1}H and ^{13}C NMR spectra of 7-hydroxyresorufinyl-dodecanoate (**1d**) Pag. 17
^{1}H and ^{13}C NMR spectra of 7-hydroxyresorufinyl-icosanoate (**1e**) Pag. 19
^{1}H and ^{13}C NMR spectra of 7-hydroxyresorufinyl-oleate (**1f**) Pag. 21
^{1}H and ^{13}C NMR spectra of 7-hydroxyresorufinyl-arachidonate (**1g**) Pag. 23
^{1}H and ^{13}C NMR spectra of 7-hydroxyresorufinyl-2-methylhexanoate (**1h**) Pag. 25
^{1}H and ^{13}C NMR spectra of 7-hydroxyresorufinyl-2-ethylhexanoate (**1i**) Pag. 27
^{1}H and ^{13}C NMR spectra of 7-hydroxyresorufinyl-2-butyloctanoate (**1j**) Pag. 29
^{1}H and ^{13}C NMR spectra of 7-hydroxyresorufinyl-2-benzoate (**1k**) Pag. 31

Table S1: Mobile phases used for the separation of synthesis products.

Compound(s)	Yields	Mobile phase	m.p.
**1a**	65%	CH_2Cl_2/acetone, 98:2	220-221 °C[a]
**1b**	71%	Petroleum ether/AcOEt gradient, from 7:3 to 5:5.	138-140 °C[a]
**1c**	68%	Petroleum ether/AcOEt 8:2.	111-112 °C
**1d**	72%	Petroleum ether/AcOEt gradient, from 8:2 to 7:3.	117-119 °C
**1e**	75%	CH_2Cl_2/acetone, 98:2	124-125 °C
**1f**	66%	Petroleum ether/AcOEt 8:2.	82 °C
**1g**	62%	CH_2Cl_2/acetone, 98:2	Oil[b]
**1h**	90%	Petroleum ether/AcOEt 75:25.	144-145 °C
**1i**	82%	CH_2Cl_2/acetone, 98:2	124-125 °C
**1j**	92%	CH_2Cl_2/acetone, 98:2	94-96 °C
**1k**	92%	Petroleum ether/ AcOEt 9:1 to remove side products, then CH_2Cl_2/acetone 98:2.	259-261 °C[a]

[a] Guilbault, G.G., and Kramer, D.N. (1965). Resorufin Butyrate and Indoxyl Acetate as Fluorogenic Substrates for Cholinesterase. *Analytical Chemistry*, *37*(1), 120-123.

[b] Lauria, S., Casati, S., and Ciuffreda, P. (2015). Synthesis and characterization of a new fluorogenic substrate for monoacylglycerol lipase and application to inhibition studies. *Analytical and Bioanalytical Chemistry*, *407*(26), 8163–8167. https://doi.org/10.1007/s00216-015-8991-9

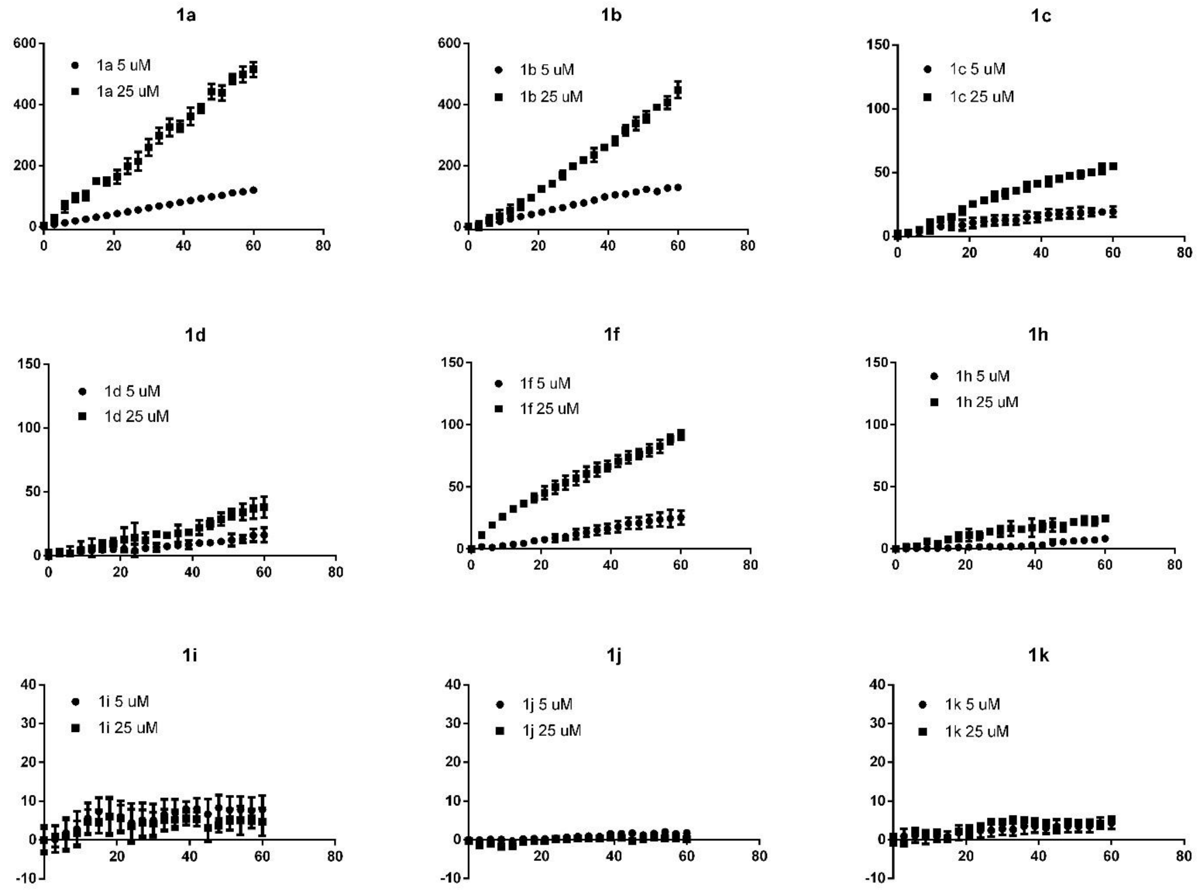

Figure S1: Comparison between spontaneous hydrolysis of **1a-d**, **1f**, and **1h-k** at lowest (circles) and highest (squares) concentrations assessed (5 μM and 25 μM). For all compounds, data have been normalized subtracting the t_0 fluorescence value from all the next fluorescence value read. Data are mean ± standard error of independent experiments.

Table S2: Ligand: MAGL interactions.

Compound	H-bond	Pi-Pi interaction
**1a**	Ala51, Met123	His269
**1b**	Ala51, Met123	His269
**1c**	Ala51	His269
**1d**	Ala51	His121, His269
**1e**	Ser122	His121, His269
**1f**	Ser122	
**1g**	Ala51, Ser122, Met123	His121, His269
**1h**	Ala51, Met123	His121, His269
**1i**	Ala51, Met123	His121, His269
**1j**	Ala51	
**1k**	Ala51	His121

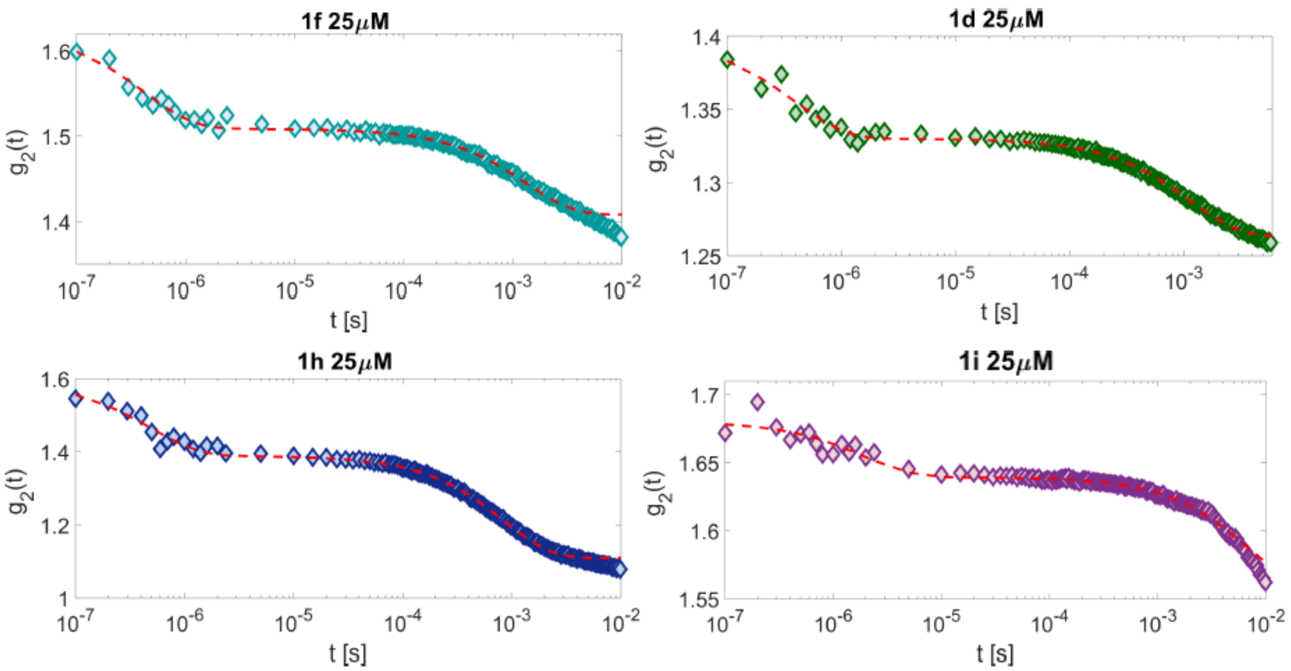

Figure S2: Time correlation functions for the scattered intensity (at θ = 90°). Red dashed lines: double exponential fit. The fastest decay time is of the order of fractions of μs which does not correspond to diffusive motion. Whereas the slower decay times reveal nano-sized particles. The corresponding decay times and radius are reported in Tab.3S.

Table S3: Decay times and corresponding radius.

SAMPLE	τ [ms]	R [nm]
**1f**	2.7±0.2	287±25
**1d**	2.3±0.2	246±16
**1h**	1.7±0.1	182±13
**1i**	13±8	1390±860

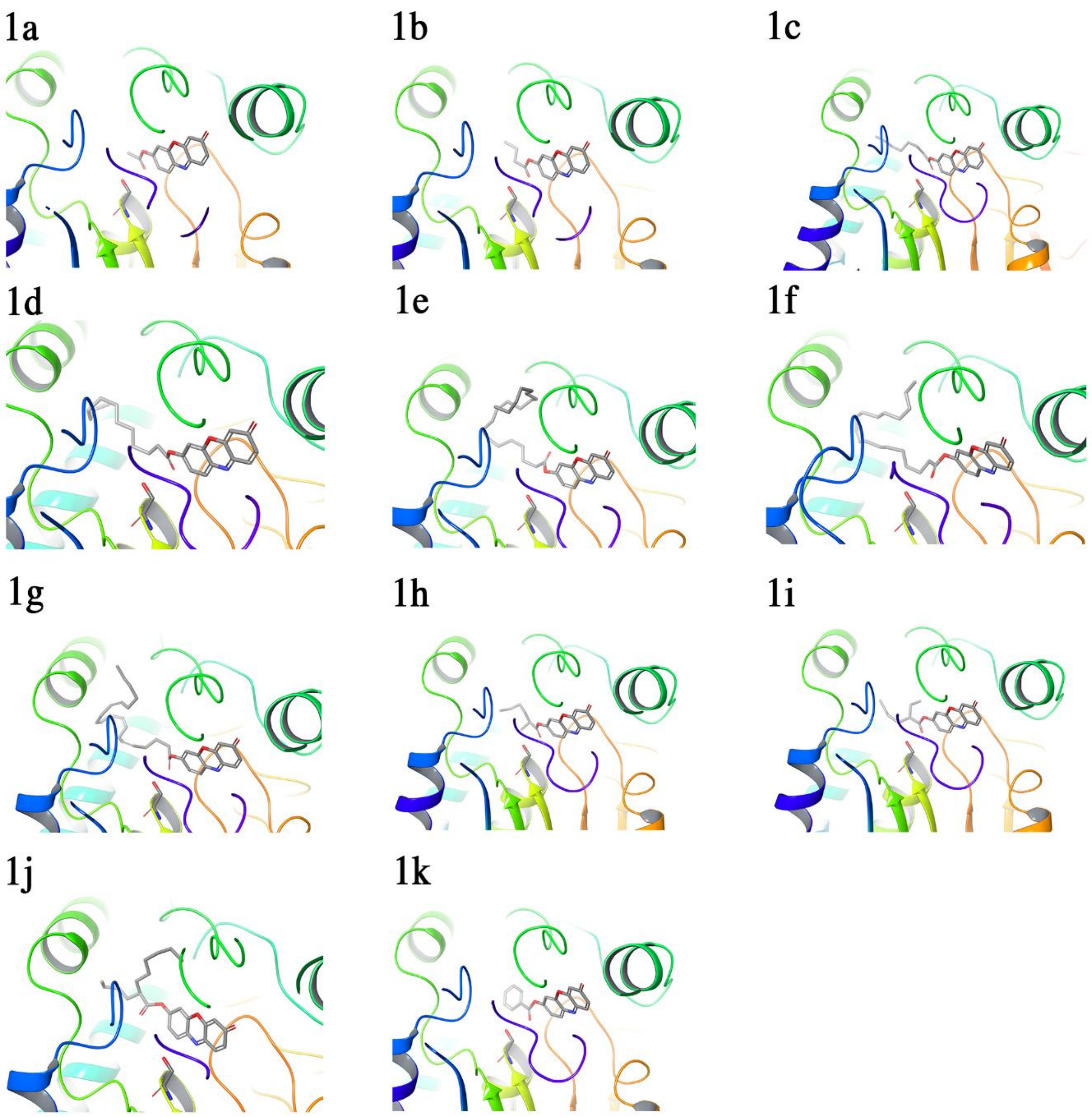

Figure S3: Binding poses of tested compounds into the MAGL binding site. MAGL is represented as ribbons; tested compounds and Ser122 are represented as sticks without hydrogen atoms.

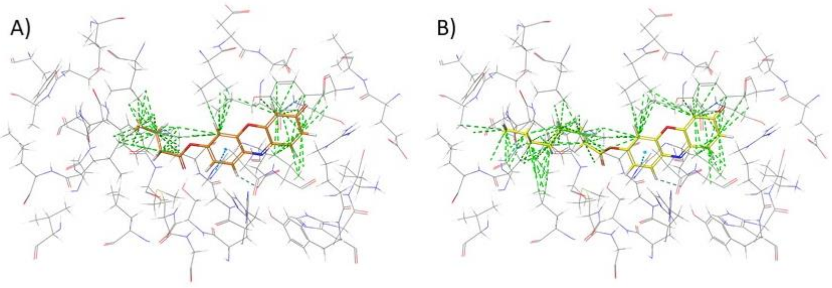

Figure S4: MAGL complexes. Compound **1b** (A) and **1c** (B) are shown in orange and yellow sticks, respectively; only MAGL residues participating to the binding site are reported in stick representation; green traces point out residues involved in hydrophobic interactions.

^{1}H and ^{13}C-NMR data

Instruments

^{1}H-NMR spectra were recorded in $CDCl_3$ (isotopic enrichment 99.95%) solutions at 300 K using a Bruker AVANCE 500 instrument (500.13 MHz for 1H, 125.76 MHz for 13C) using 5 mm inverse detection broadband probes and deuterium lock. Chemical shifts (d) are given as parts per million relative to the residual solvent peak (7.26 ppm for 1H) and coupling constants (J) are in Hertz. The experimental error in the measured ^{1}H-^{1}H coupling constants is ± 0.5 Hz. The splitting pattern abbreviations are as follows: s, singlet; d, doublet; t, triplet; q, quartet; m, multiplet, and bs, broad peak. For two-dimensional experiments, Bruker microprograms using gradient selection (gs) were applied.

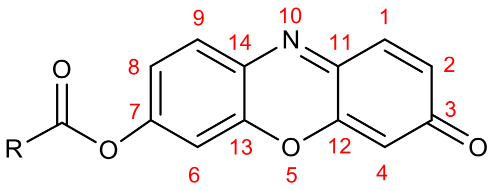

Figure S5. IUPAC numbering of 7-hydroxyresorufinyl moiety.

Table S4. Assignment of ^{1}H and ^{13}C-NMR Chemical Shift in $CDCl_3$.

	1a		***1c***		***1b***		***1f***		***1k***		***1d***	
	δ (H)	δ (C)	δ (H)	δ (C)	δ (H)	δ (C)	δ (H)	δ (C)	δ (H)	δ (C)	δ (H)	δ (C)
CH(1)	7.46 (d)<br>*9.8*	134.8	7.47<br>*9.8*	134.9	7.46<br>*9.9*	134.8	7.46<br>*9.8*	134.8	7.49<br>*9.8*	134.8	7.47<br>*9.8*	134.8
CH(2)	6.89 (dd)<br>*9.8, 2.0*	135.2	6.90<br>*9.8, 2.0*	135.1	6.89<br>*9.9, 2.1*	135.1	6.90<br>*9.8, 2.0*	135.1	6.91<br>*9.8, 2.0*	135.2	6.90<br>*9.8, 2.0*	135.1
C(3)		186.3		186.3		186.3		186.3		186.3		186.3
CH(4)	6.36 (d)<br>*2.0*	107.2	6.37<br>*2.0*	107.2	6.36<br>*2.1*	107.2	6.36<br>*2.0*	107.2	6.38<br>*2.0*	107.3	6.37<br>*2.0*	107.2
CH(6)	7.18 (d)<br>*2.4*	109.7	7.18<br>*2.4*	109.7	7.18<br>*2.4*	109.7	7.17<br>*2.4*	109.7	7.32<br>*2.4*	109.9	7.18<br>*2.4*	109.7
C(7)		153.5		153.7		153.6		153.7		153.8		153.7
CH(8)	7.15 (dd)<br>*8.6, 2.4*	119.3	7.15<br>*8.6, 2.4*	119.3	7.15<br>*8.6, 2.4*	119.3	7.15<br>*8.6, 2.4*		7.30<br>*8.6, 2.4*	119.4	7.15<br>*8.6, 2.4*	119.3
CH(9)	7.82 (d)<br>*8.6*	131.1	7.83<br>*8.6*	131.1	7.82<br>*8.6*	131.1	7.83<br>*8.6*		7.89<br>*8.6*	134.2	7.83<br>*8.6*	131.1
C(11)		148.3		148.2		148.2		148.2		148.3		148.2
C(12)		149.3		149.3		149.3		149.3		149.3		149.3
C(13)		144.3		144.4		144.3		144.4		144.5		144.4
C(14)		131.2		131.2		131.1		131.2		131.2		131.2
COO		168.5		171.4		171.2		171.4		164.4		171.4
OCO*CH*$_2$			2.63 (t)<br>*7.5*	34.4	2.62<br>*7.0*	36.2	2.63<br>*7.0*	34.4			2.63<br>*7.5*	34.4
CO<br>CH_2*CH*$_2$			1.79 (tt)<br>*7.5, 7.5*	31.6	1.84<br>*7.0, 7.4*	18.3	1.80<br>*7.0, 7.0*	22.7			1.79<br>*7.5, 7.5*	24.8
CH=CH							5.42-5.34<br>(2H, m,<br>9′, 10′)	129.7 (9′),<br>130.1 (10′)				

CH=CH-*CH_2*							2.08-2.02 (4H, m, 8′, 11′)	27.1 (8′), 27.2 (11′)				
CH_2-FAC[a]			1.27-1.47 (20 H, m, 4′-7′)	29.0, 28.9, 24.8, 24.7			1.45-1.26 (20 H, m, 17′)	22.7 (17′), 29.0, 29.1, 29.2, 29.4, 29.5, 29.6, 29.7 (4′-7′ and 12′-15′), 31.9 (16′)			1.47-1.24 (16 H, m, 4′-11′)	22.7, 29.1, 29.2, 29.3, 29.5, 29.6, 31.9 (4′-11′)
CH_3	2.38	21.2	0.93 *7.0*	14.1	1.09 *7.4*	13.6		14.1				
o-H_{ar}									8.24 (d) *7.7*	130.4		
m-H_{ar}									7.58 (t) *7.7*	128.8		
p-H_{ar}									7.72 (t) *7.7*	131.2		

[a]fatty acid chain

Continued from Table S4…

	1j		***1h***		***1i***		***1e***		***1g***	
	δ (H)	δ (C)	δ (H)	δ (C)	δ (H)	δ (C)	δ (H)	δ (C)	δ (H)	δ (C)
CH(1)	7.46 (d)<br>*9.8*	134.8	7.46 (d)<br>*9.8*	134.8	7.47 (d)<br>*9.8*	134.8	7.46<br>*9.8*	134.8	7.46<br>*9.8*	134.8
CH(2)	6.89 (dd)<br>*9.8, 2.0*	135.1	6.89 (dd)<br>*9.8, 2.0*	135.1	6.90<br>(dd)<br>*9.8, 2.0*	135.1	6.89<br>*9.8, 2.0*	135.1	6.89<br>*9.8, 2.0*	135.2
C(3)		186.3		186.3		186.3		186.3		186.3
CH(4)	6.36 (d)<br>*2.0*	107.2	6.36 (d)<br>*2.0*	107.2	6.36 (d)<br>*2.0*	107.2	6.36<br>*2.0*	107.2	6.36<br>*2.0*	107.3
CH(6)	7.15 (d)<br>*2.4*	109.7	7.16 (d)<br>*2.4*	109.7	7.16 (d)<br>*2.4*	109.8	7.18<br>*2.4*	109.7	7.17<br>*2.4*	109.7
C(7)		153.8		153.8		153.8		153.7		153.6
CH(8)	7.13 (dd)<br>*8.6, 2.4*	119.4	7.14 (dd)<br>*8.6, 2.4*	119.3	7.13<br>(dd)<br>*8.6, 2.4*	119.4	7.14<br>*8.6, 2.4*	119.3	7.14<br>*8.6, 2.4*	
CH(9)	7.83 (d)<br>*8.6*	131.1	7.83 (d)<br>*8.6*	131.1	7.83 (d)<br>*8.6*	131.1	7.82<br>*8.6*	131.1	7.82<br>*8.6*	
C(11)		148.2		148.2		148.2		148.2		148.3
C(12)		149.3		149.4		149.3		149.3		149.4
C(13)		144.3		144.4		144.4		144.4		144.3
C(14)		131.2		131.2		131.1		131.2		131.2
COO		174.2		174.5		174.1		171.4		
OCO*CH*$_{(2,1)}$(CH$_2$)$_n$	2.63 (tt)<br>*7.0, 7.0*	45.9	2.75 (tq)<br>*7.0*	39.7	2.58 (tt)<br>*7.7, 7.0*	47.4	2.63<br>*7.5*	34.4	2.65 (t)<br>*7.0*	33.7 (2′)
OCOCH$_{(2,1)}$(*CH*$_2$)$_n$	1.83-1.74<br>(m)<br>1.67-1.59<br>(m)	32.4<br>32.1	1.84 (3′)<br>*7.0, 7.0*	33.3	1.80 (3′)<br>*7.4, 7.0*	33.3	1.79<br>*7.5, 7.5*	24.8	1.88 (3′)<br>*7.0, 7.3*	24.6 (3′)

CH=CH				-		-			5.52-5.33 (8H, m, 5′,6′,8′,9′, 11′,12′, 14′,15′)	130.6,129.5,128.7, 128.5,128.4,128.1,127.8,127.5 (5′,6′,8′,9′, 11′,12′, 14′,15′)
CH=CH-*CH_2*				-		-			2.88-2.82 (6H, m, 7′, 10′, 13′)	27.7 (7′, 10′, 13′)
CH_2-FAC[a]	1.44-1.28 (12 H, m)	31.7, 29.7, 29.2, 27.5, 22.6, 22.6	1.64-1.58 1.45-1.39 (4H)	29.4 22.6	1.74-1.62 1.46-1.36 (6H)	31.6 29.6 25.4	1.46-1.25 (32 H, m, 4′-19′)	22.7, 248, 29.1, 29.2, 29.3, 29.5, 29.6, 29.7, 31.9 (4′-19′)	2.25 (4′, dt) *7.0, 7.3* 1.39-1.28 (6 H, m, 17′-19′)	22.6 (19′), 29.3 (17′), 31.5 (18′), 26.5 (4′), 27.2 (16′)
CH_3	0.96 (t) 0.92 (t) *7.0*	14.1 14.0	1.34 (d) 0.97 (t) *7.0*	10.4 16.9	1.06 (t) *7.4* 0.97 (t) *7.0*	11.9 14.0	0.90 (t) *7.0*	14.1	0.91 (t) *7.6*	14.1

^{1}H and ^{13}C NMR spectra of 7-hydroxyresorufinyl-acetate (**1a**)

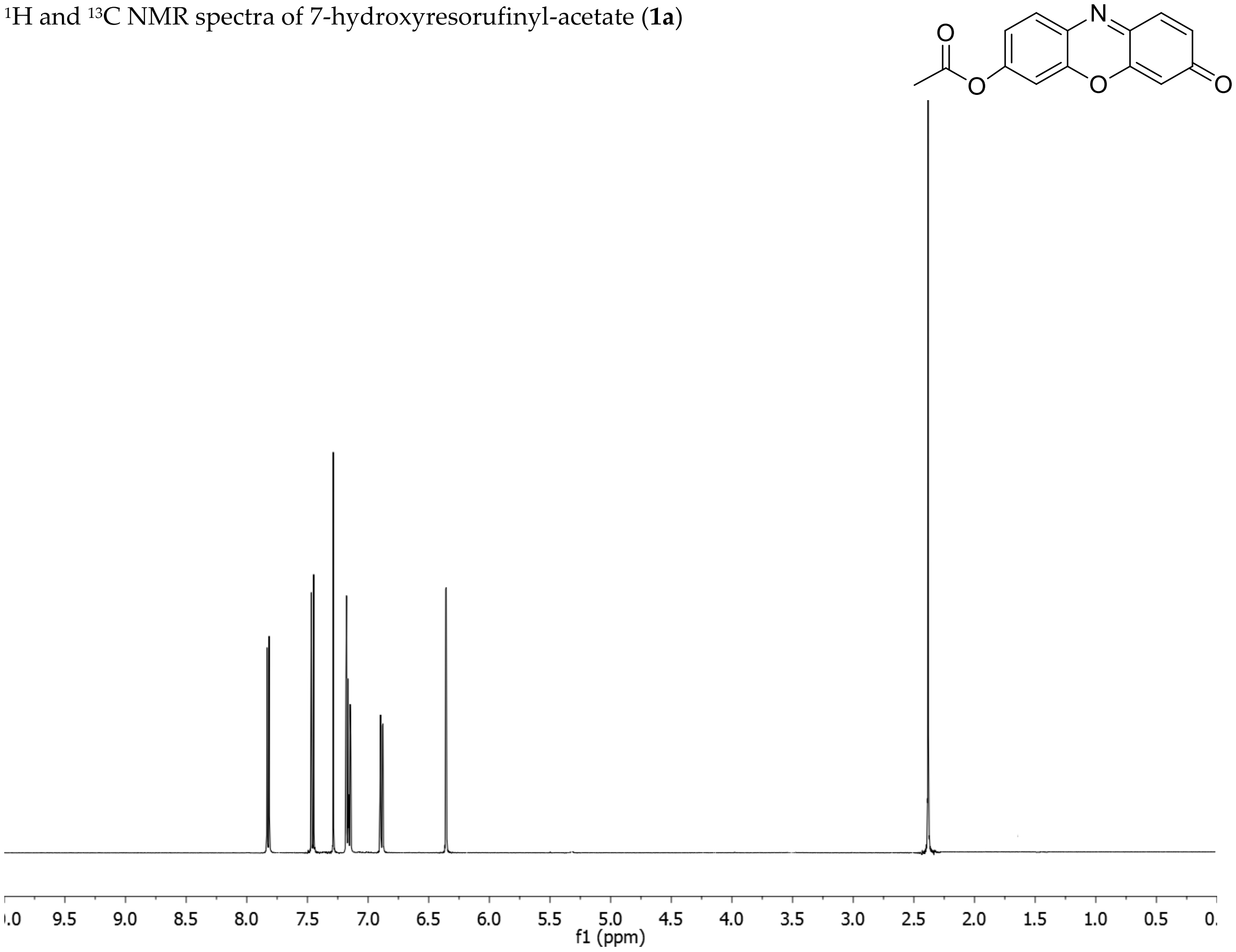

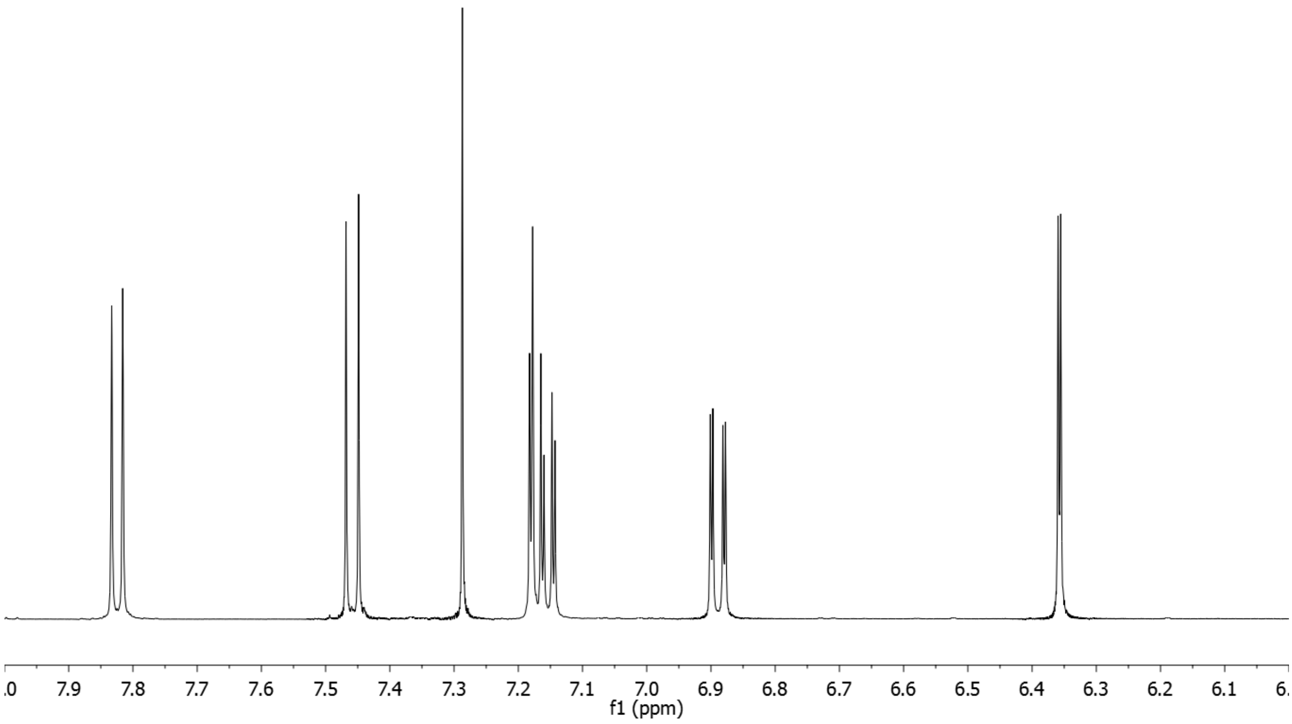

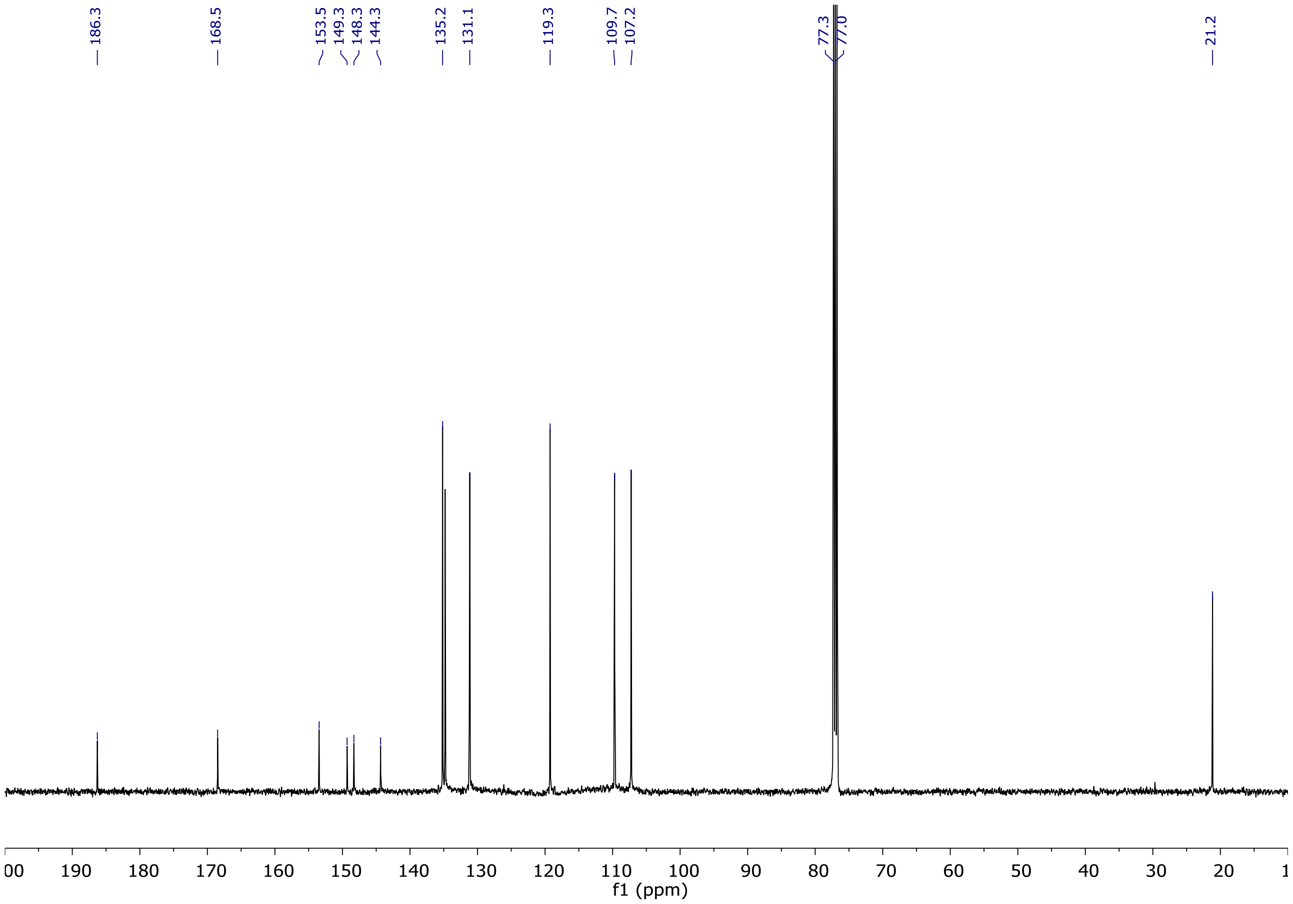
186.3
168.5
153.5
149.3
148.3
144.3
135.2
131.1
119.3
109.7
107.2
77.3
77.0
21.2
f1 (ppm)

^{1}H and ^{13}C NMR spectra of 7-hydroxyresorufinyl-butyrate (**1b**)

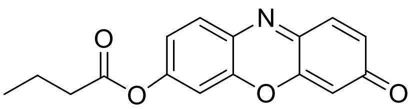

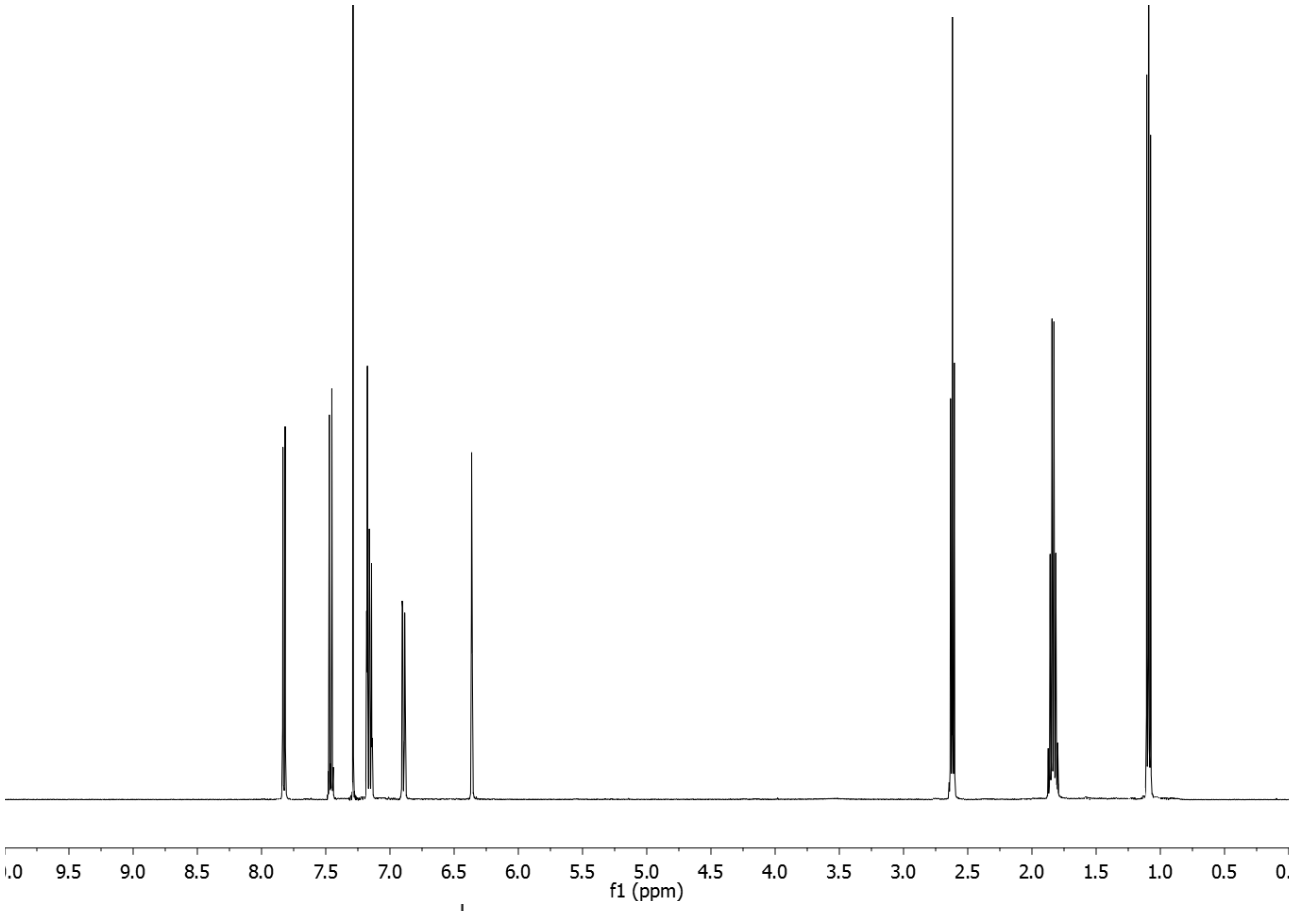

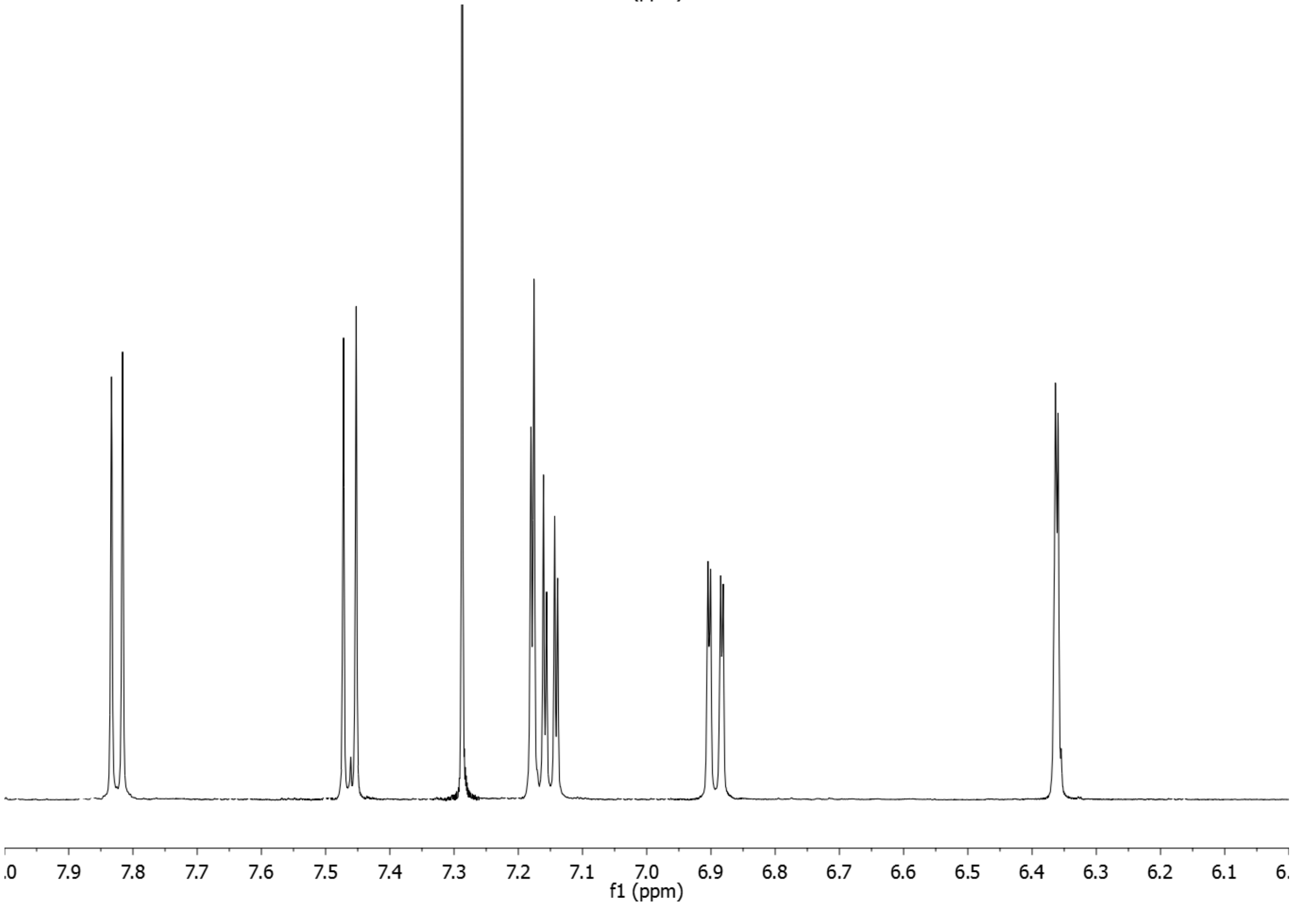

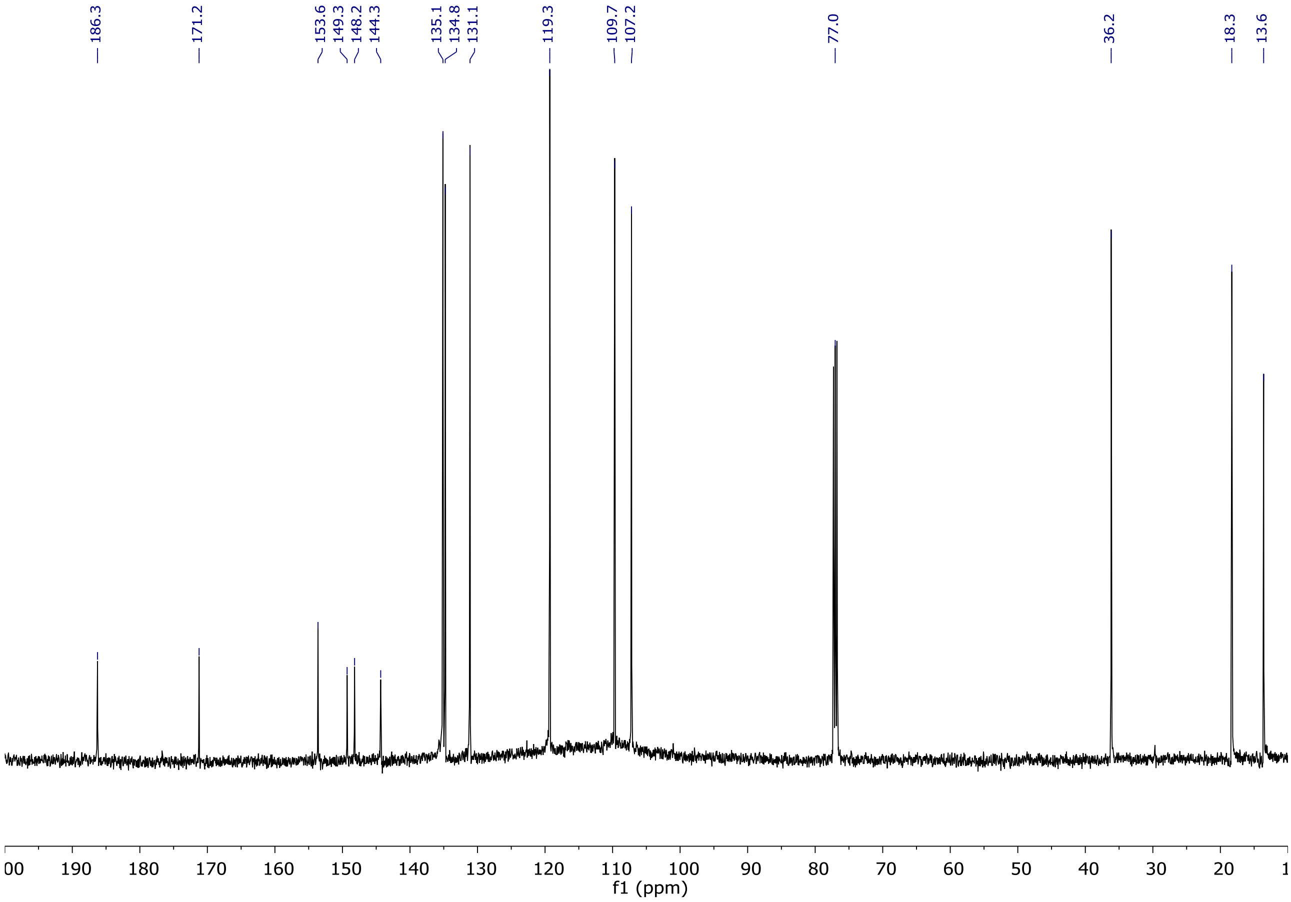
186.3
171.2
153.6
149.3
148.2
144.3
135.1
134.8
131.1
119.3
109.7
107.2
77.0
36.2
18.3
13.6
f1 (ppm)

^{1}H and ^{13}C NMR spectra of 7-hydroxyresorufinyl-octanoate (**1c**)

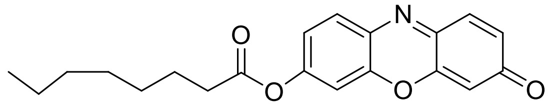

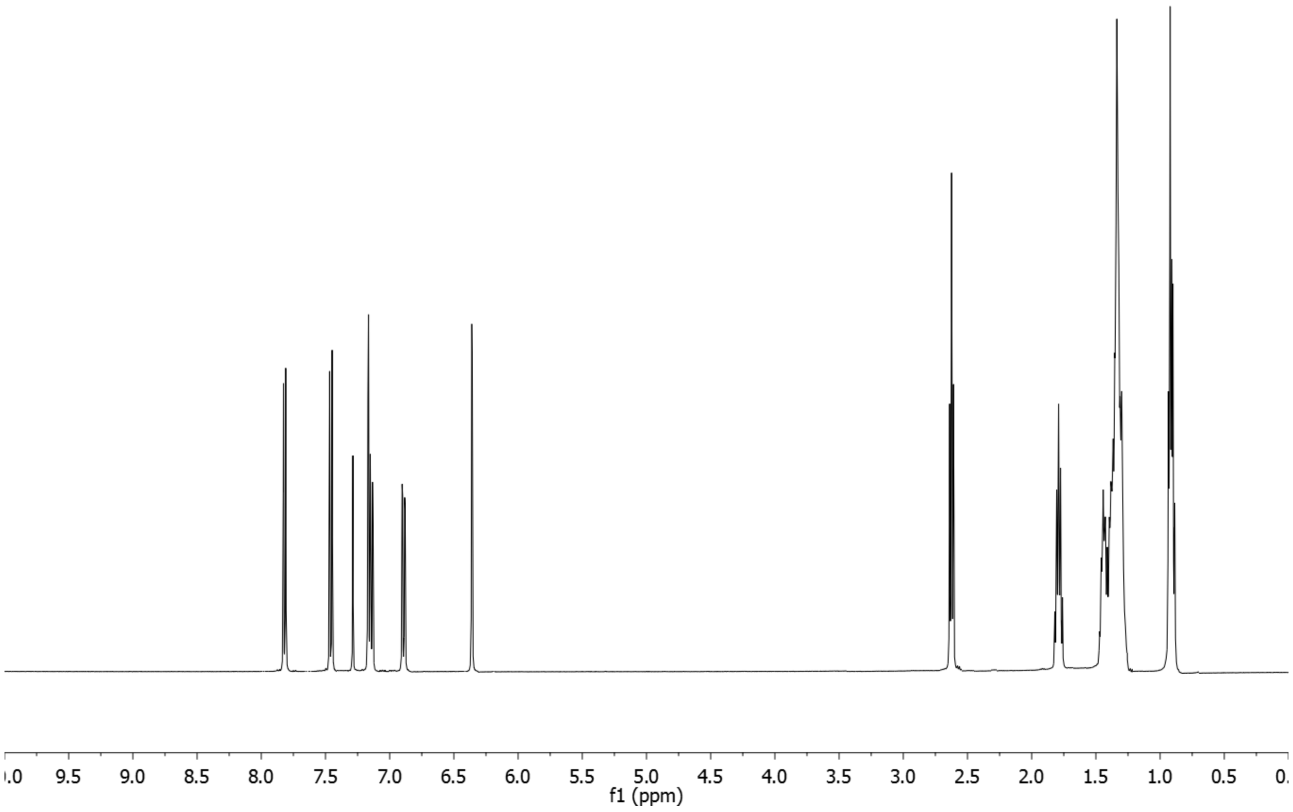

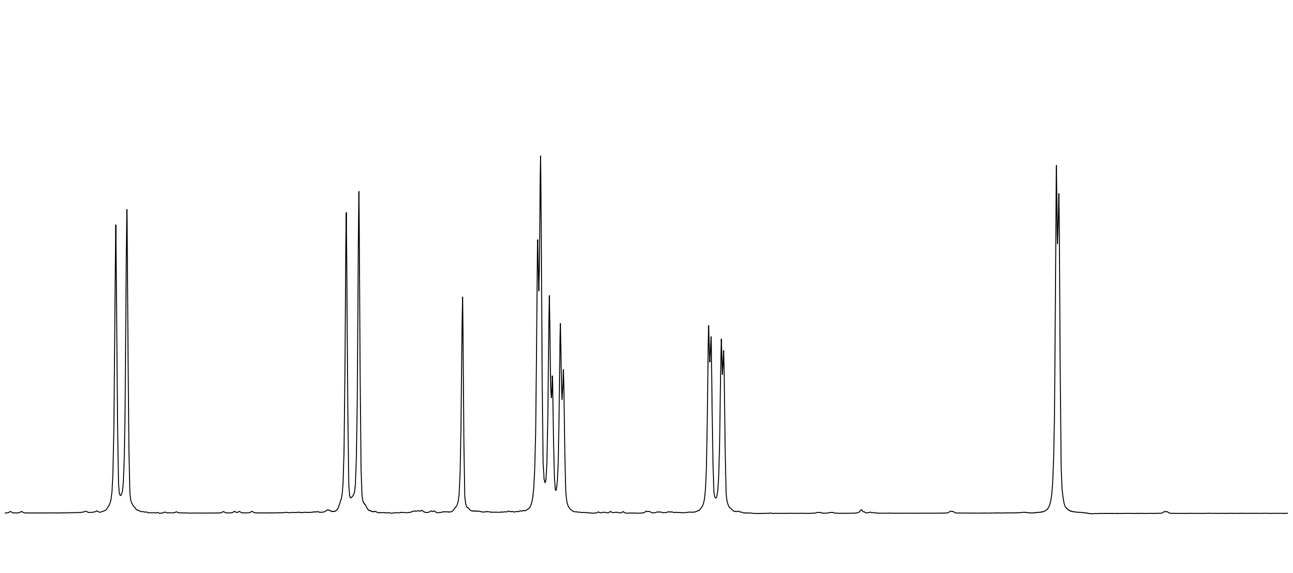

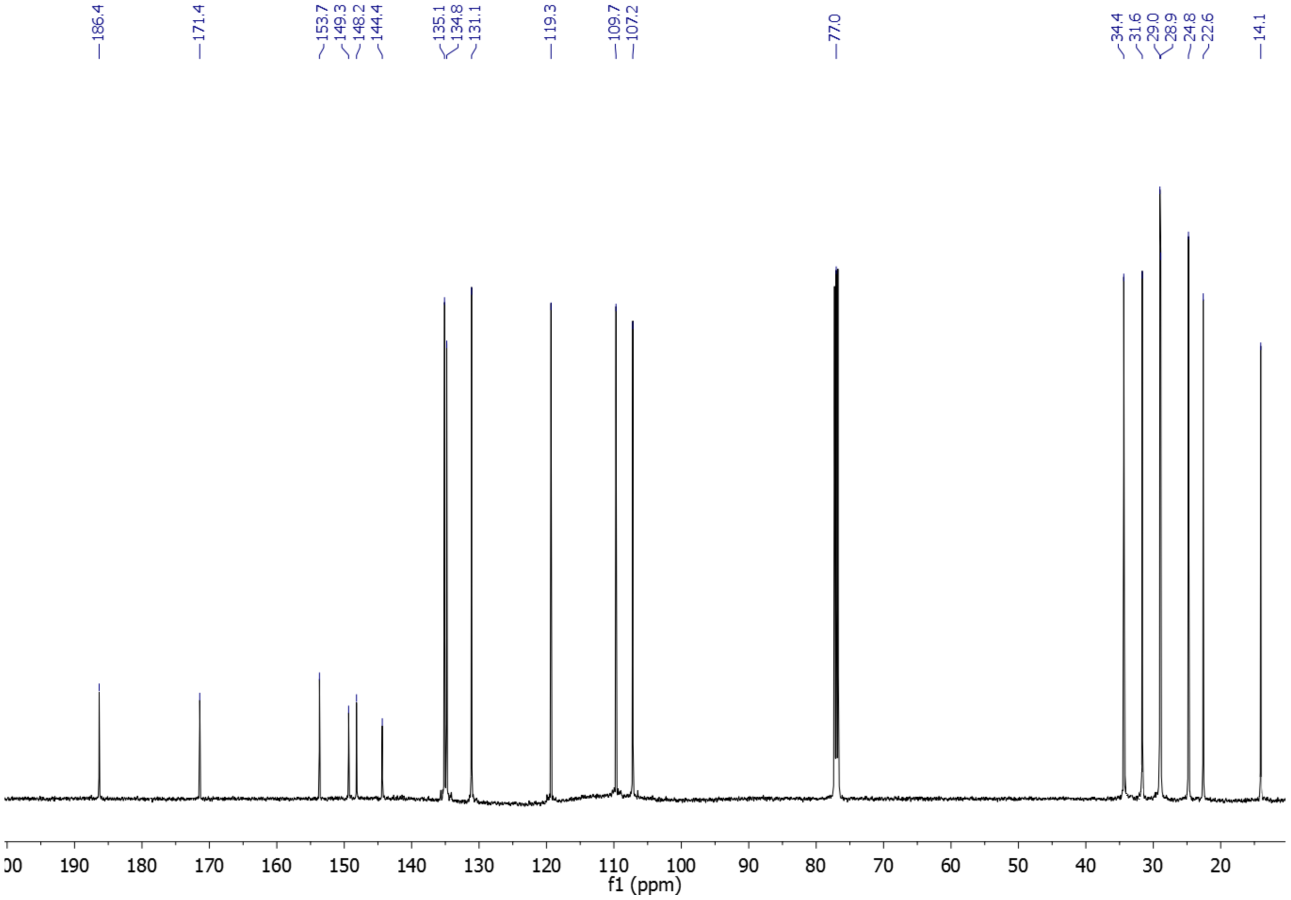
186.4
171.4
153.7
149.3
148.2
144.4
135.1
134.8
131.1
119.3
109.7
107.2
77.0
34.4
31.6
29.0
28.9
24.8
22.6
14.1
f1 (ppm)

^{1}H and ^{13}C NMR spectra of 7-hydroxyresorufinyl-dodecanoate (**1d**)

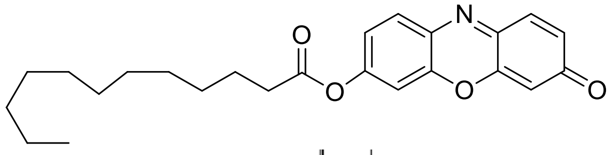

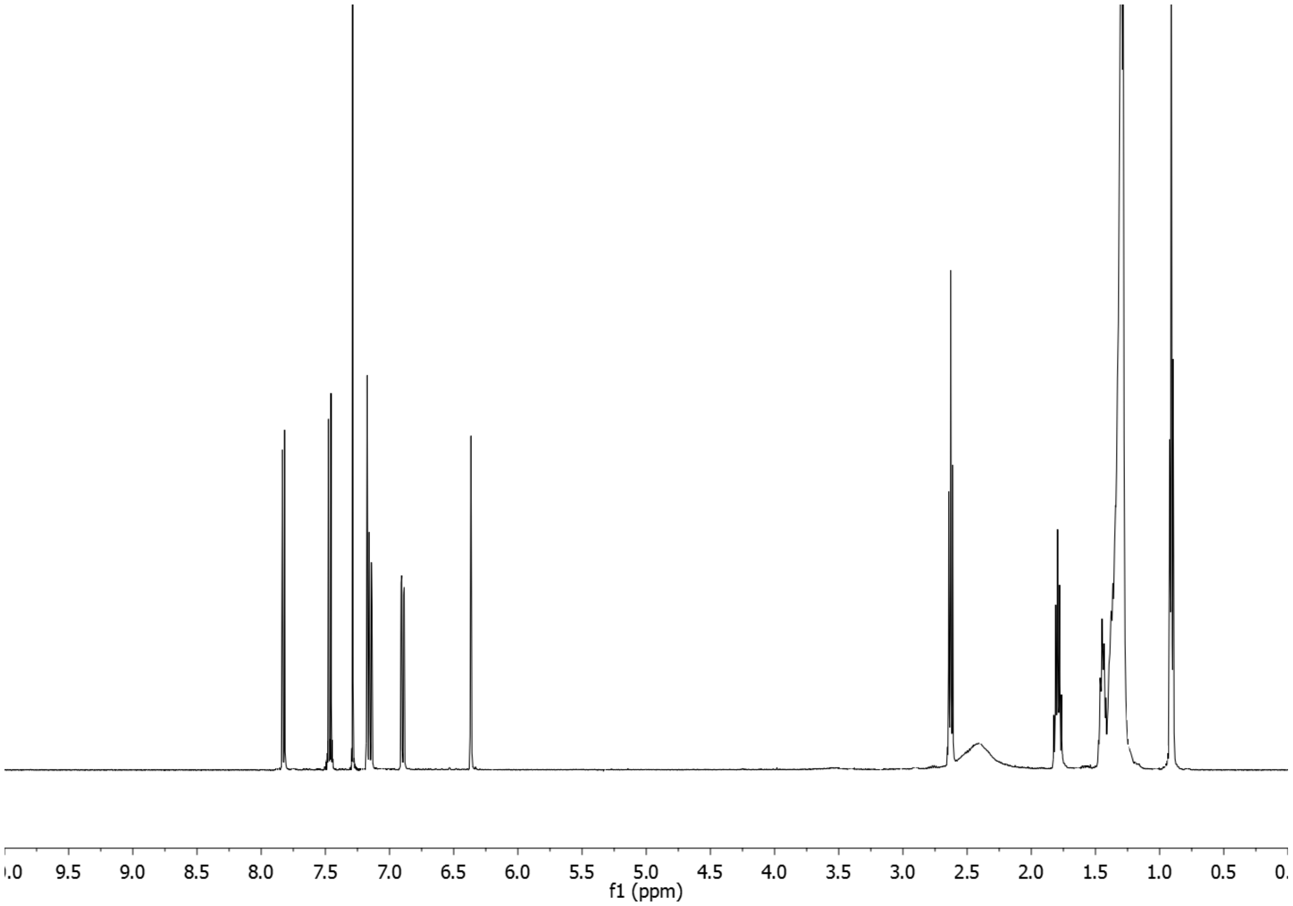

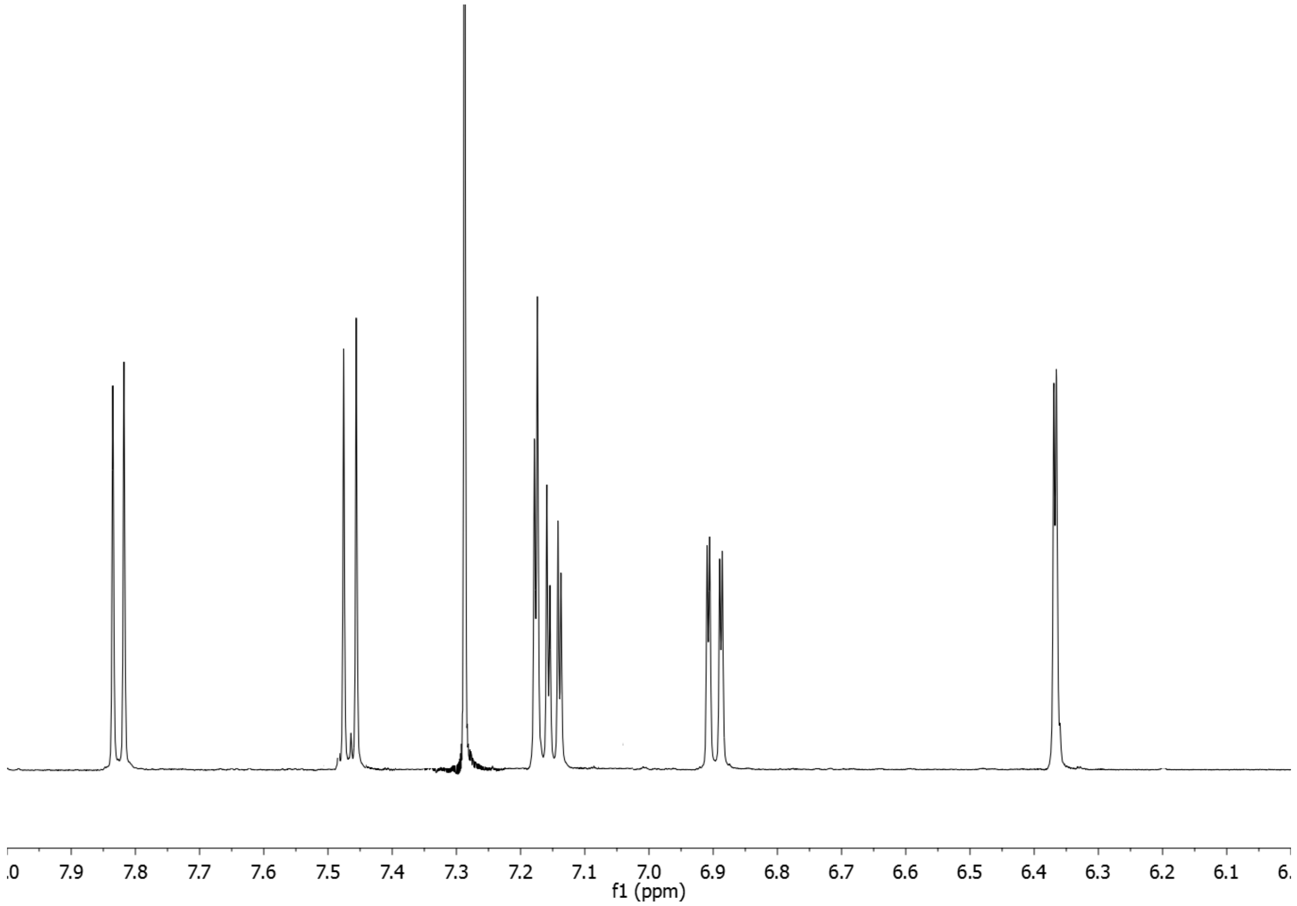

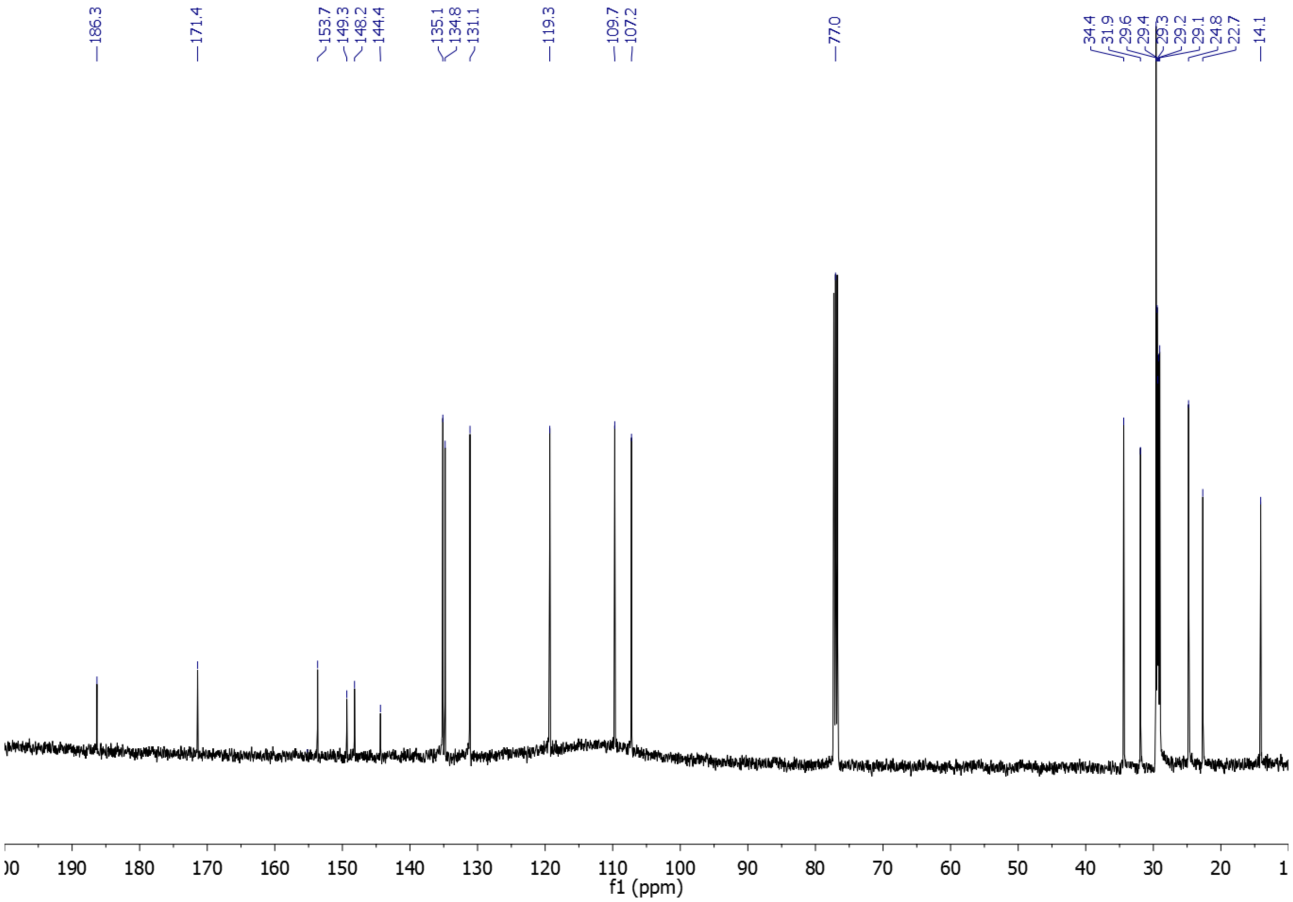
186.3
171.4
153.7
149.3
148.2
144.4
135.1
134.8
131.1
119.3
109.7
107.2
77.0
34.4
31.9
29.6
29.4
29.3
29.2
29.1
24.8
22.7
14.1
190
180
170
160
150
140
130
120
110
100
90
80
70
60
50
40
30
20
f1 (ppm)

^{1}H and ^{13}C NMR spectra of 7-hydroxyresorufinyl-icosanoate (**1e**)

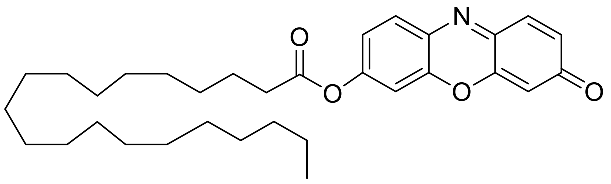

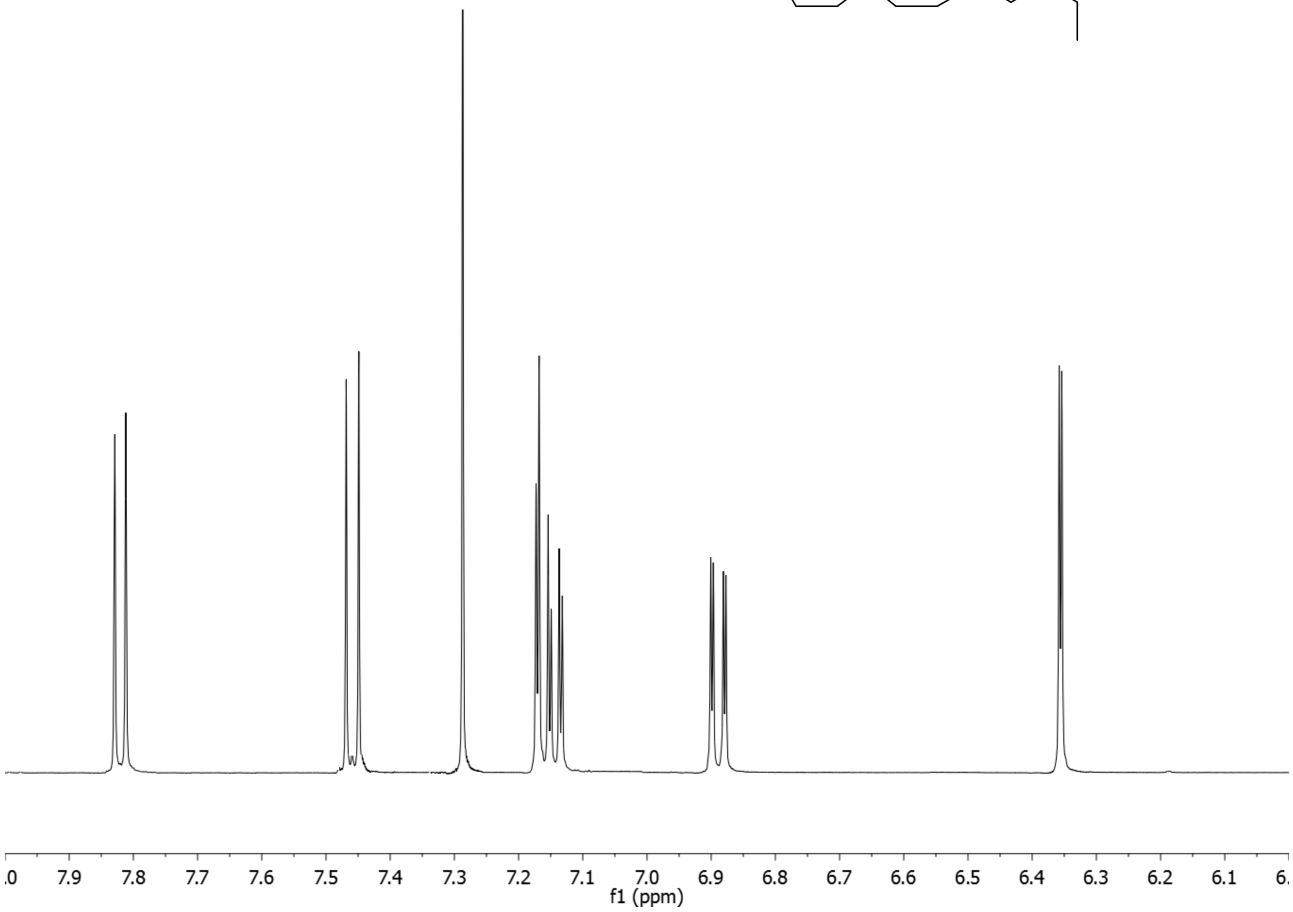

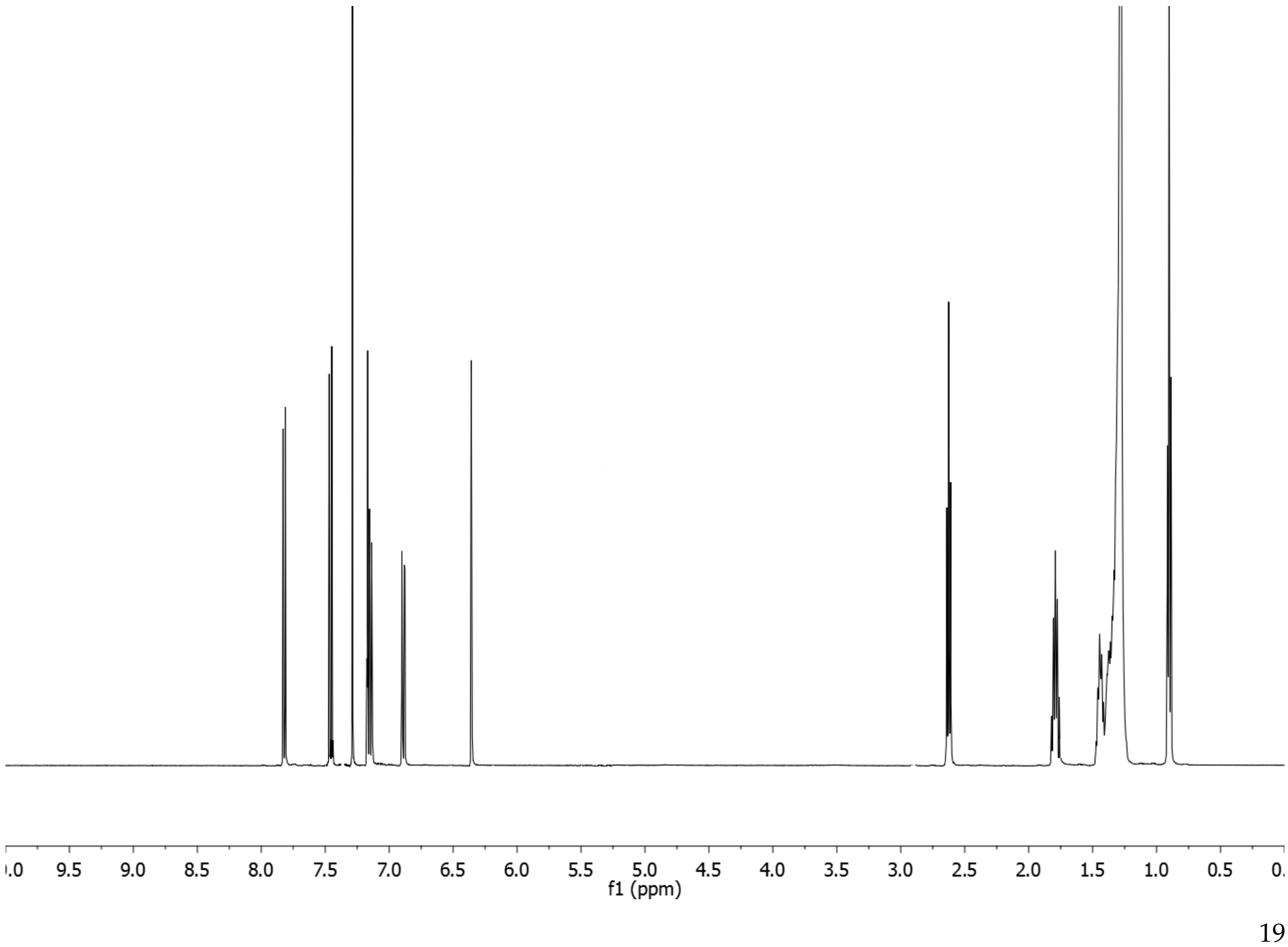

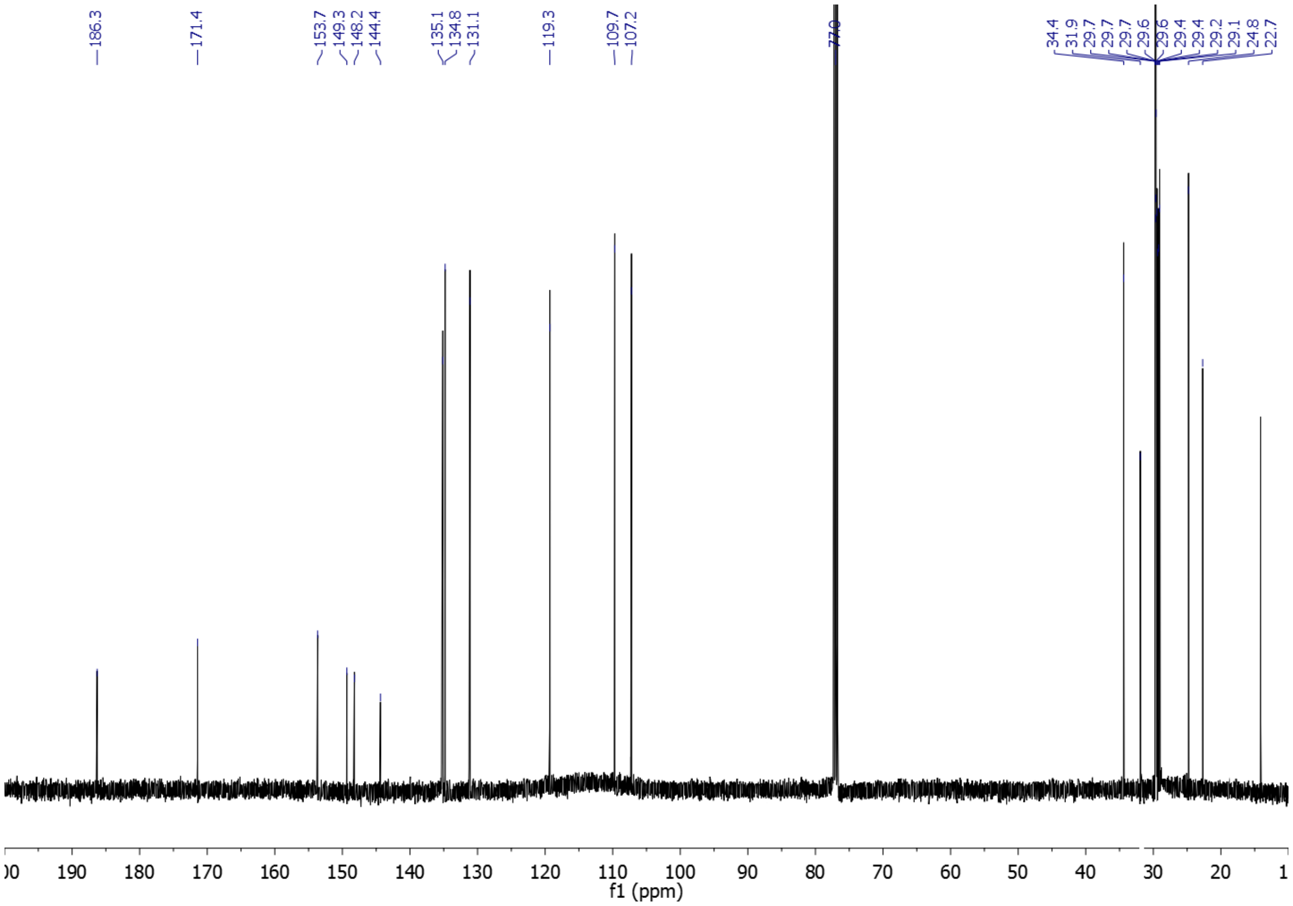
186.3
171.4
153.7
149.3
148.2
144.4
135.1
134.8
131.1
119.3
109.7
107.2
77.0
34.4
31.9
29.7
29.7
29.7
29.6
29.6
29.4
29.4
29.2
29.1
24.8
22.7
f1 (ppm)

^{1}H and ^{13}C NMR spectra
of 7-hydroxyresorufinyl-oleate (**1f**)

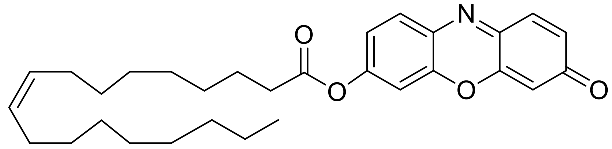

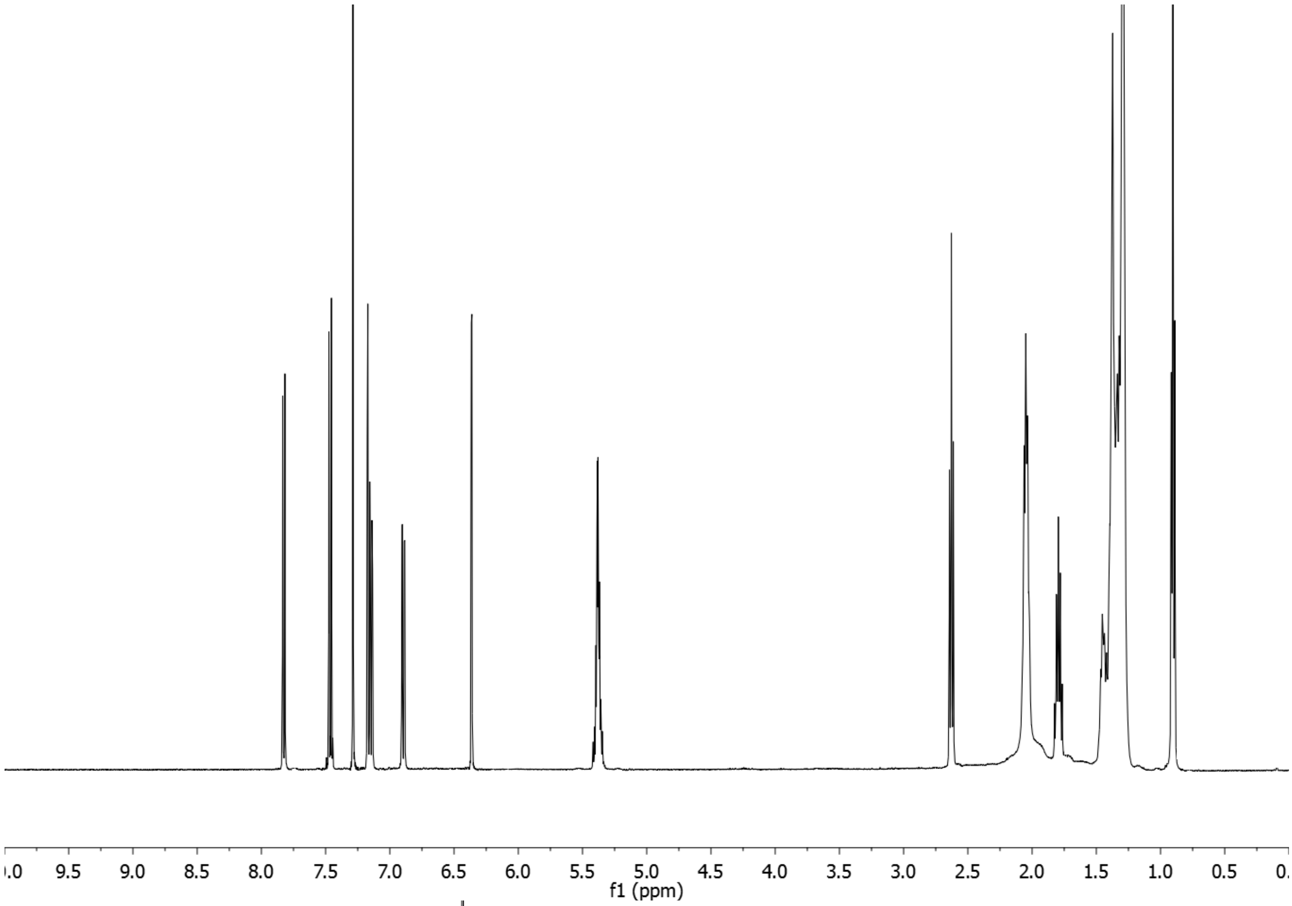

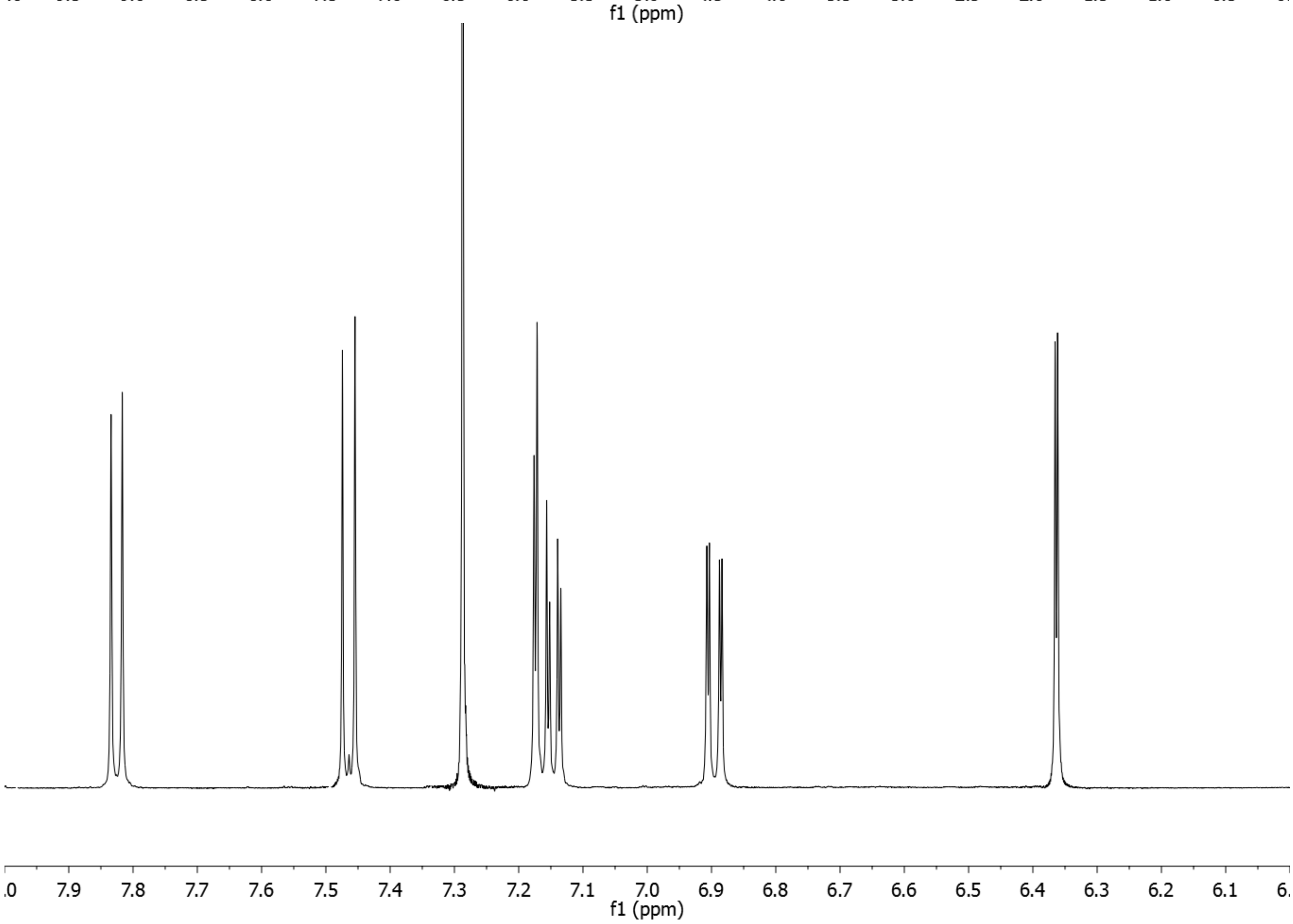

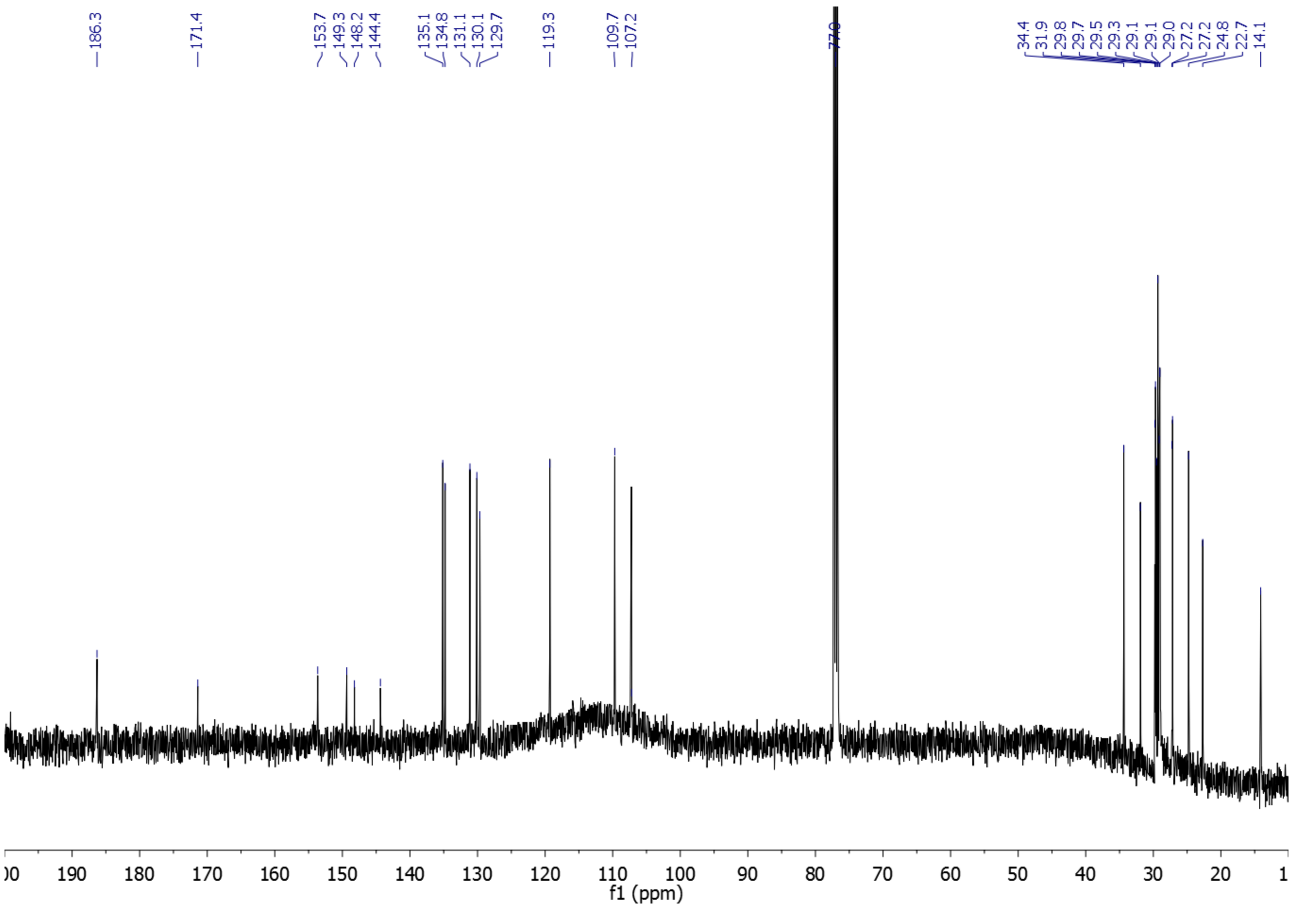

186.3
171.4
153.7
149.3
148.2
144.4
135.1
134.8
131.1
130.1
129.7
119.3
109.7
107.2
77.0
34.4
31.9
29.8
29.7
29.5
29.3
29.1
29.1
29.0
27.2
27.2
24.8
22.7
14.1
f1 (ppm)

^{1}H and ^{13}C NMR spectra of 7-hydroxyresorufinyl-arachidonate (**1g**)

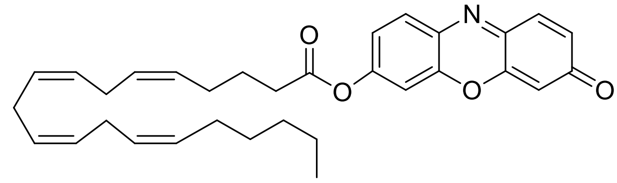

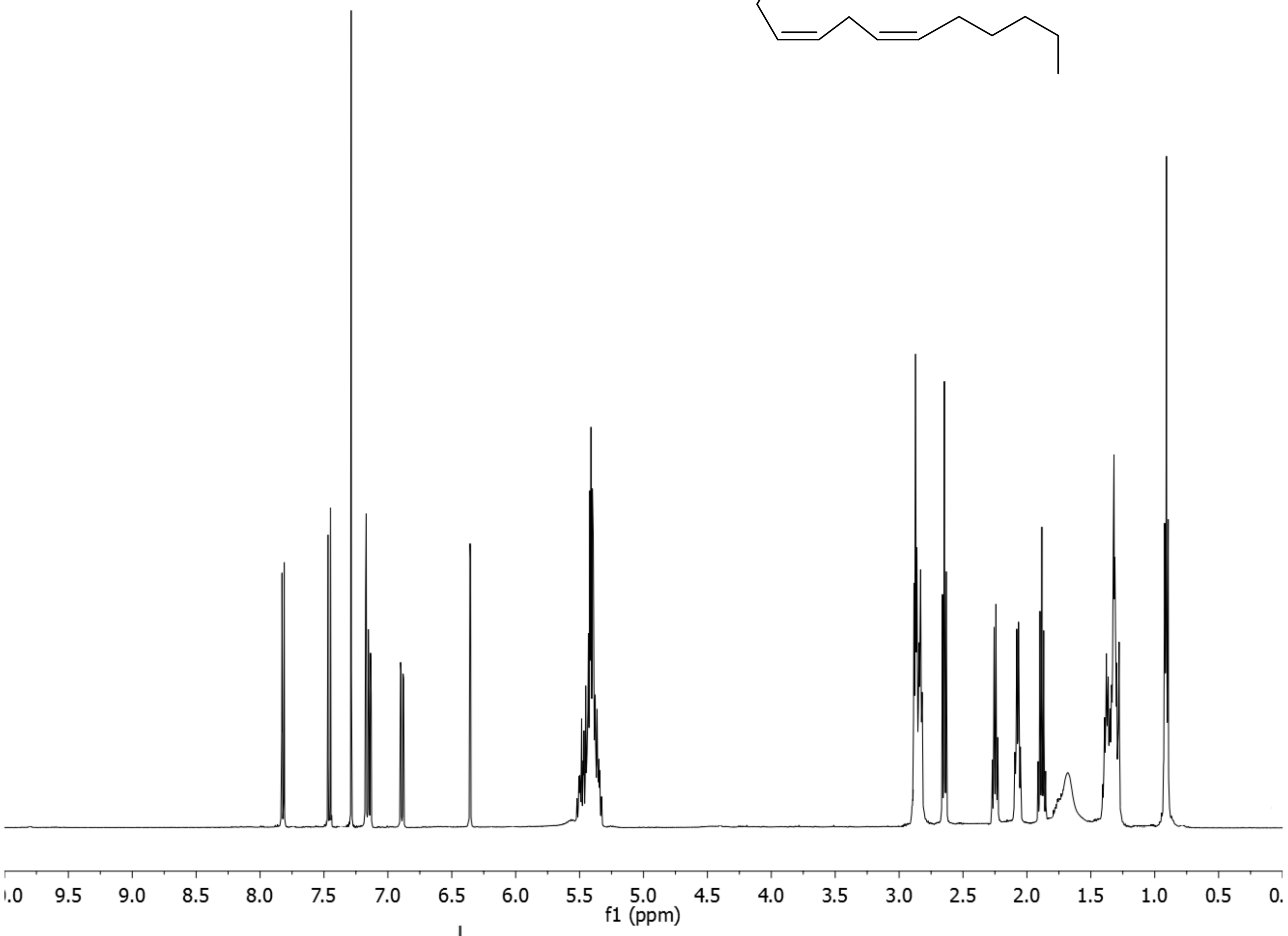

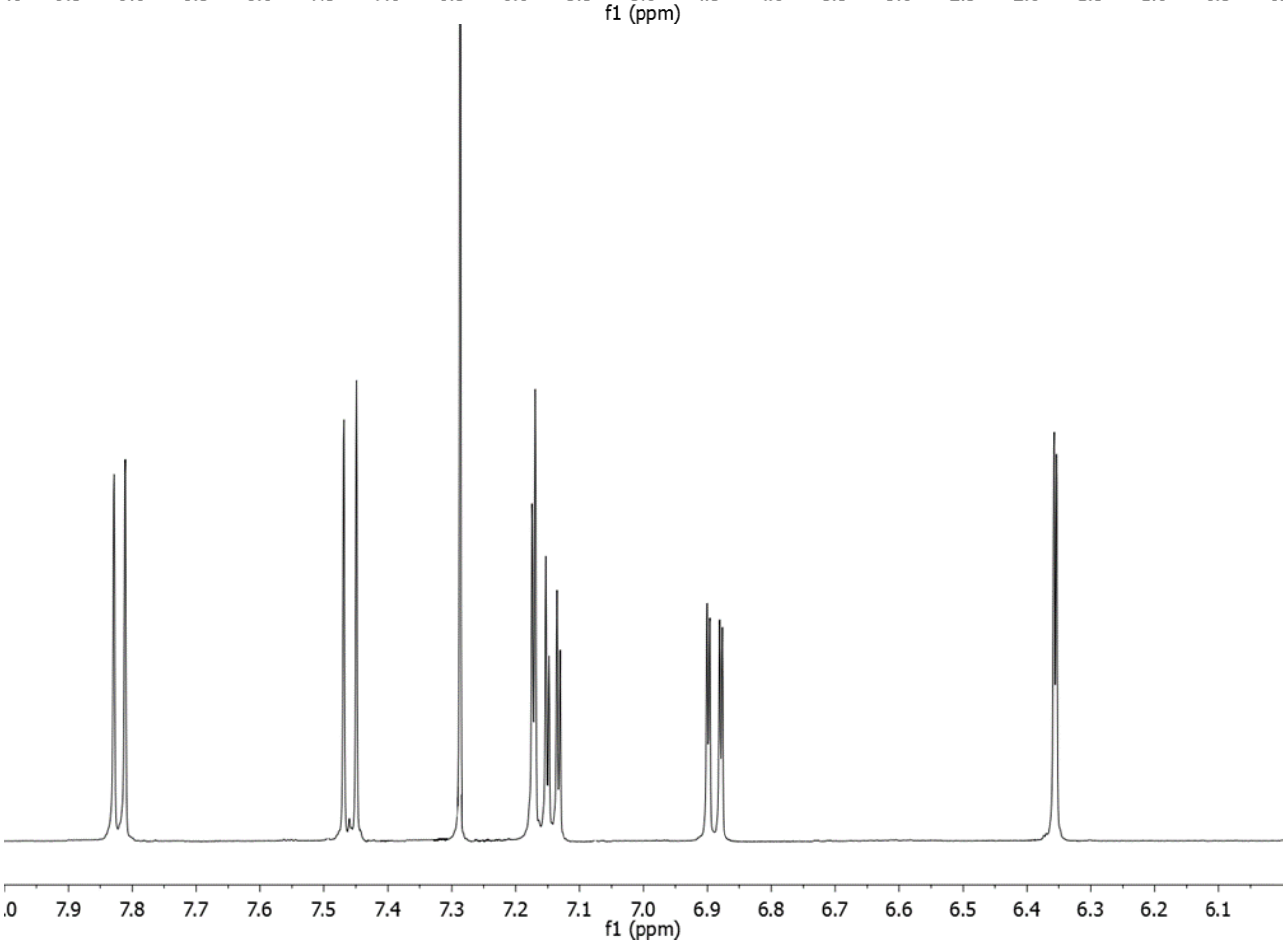

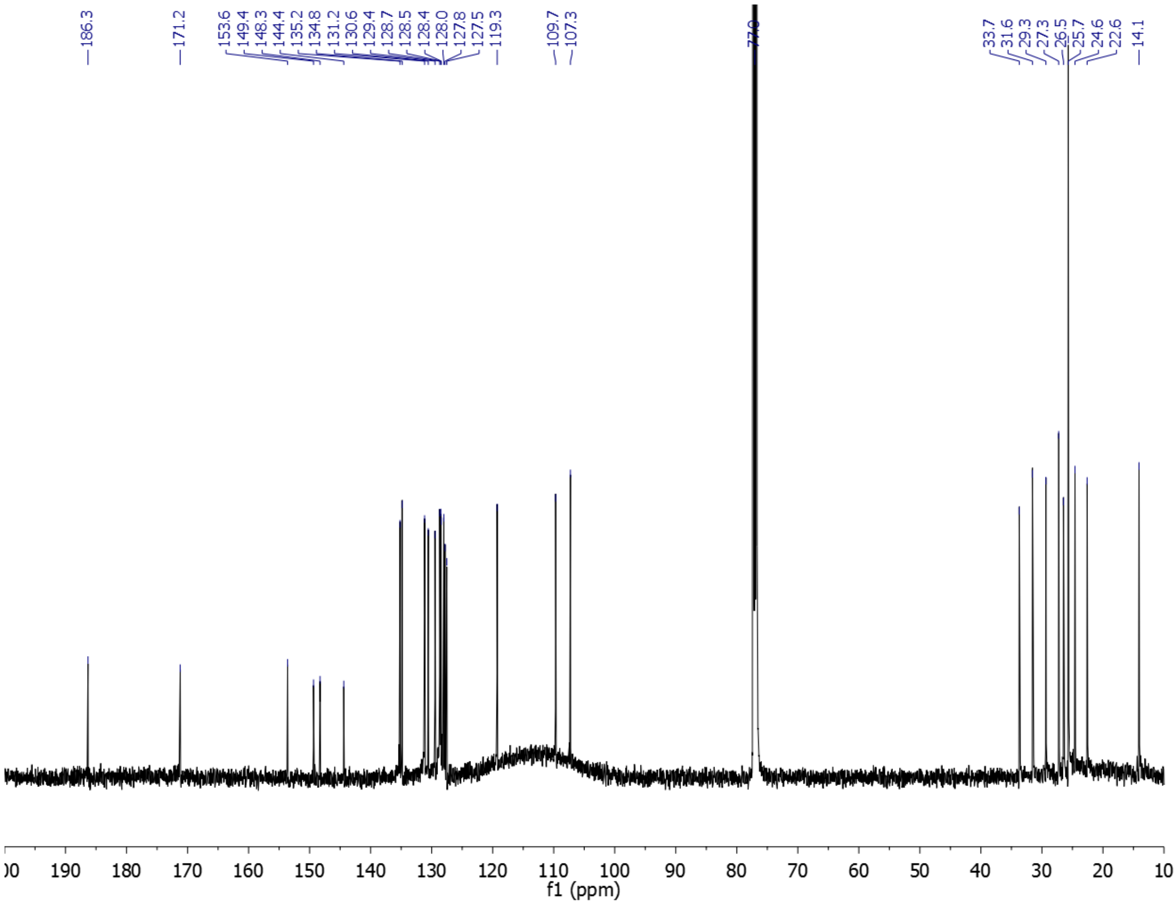
186.3
171.2
153.6
149.4
148.3
144.4
135.2
134.8
131.2
130.6
129.4
128.7
128.5
128.4
128.0
127.8
127.5
119.3
109.7
107.3
77.0
33.7
31.6
29.3
27.3
26.5
25.7
24.6
22.6
14.1
f1 (ppm)

^{1}H and ^{13}C NMR spectra of 7-hydroxyresorufinyl-2-methylhexanoate (**1h**)

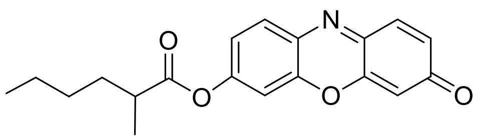

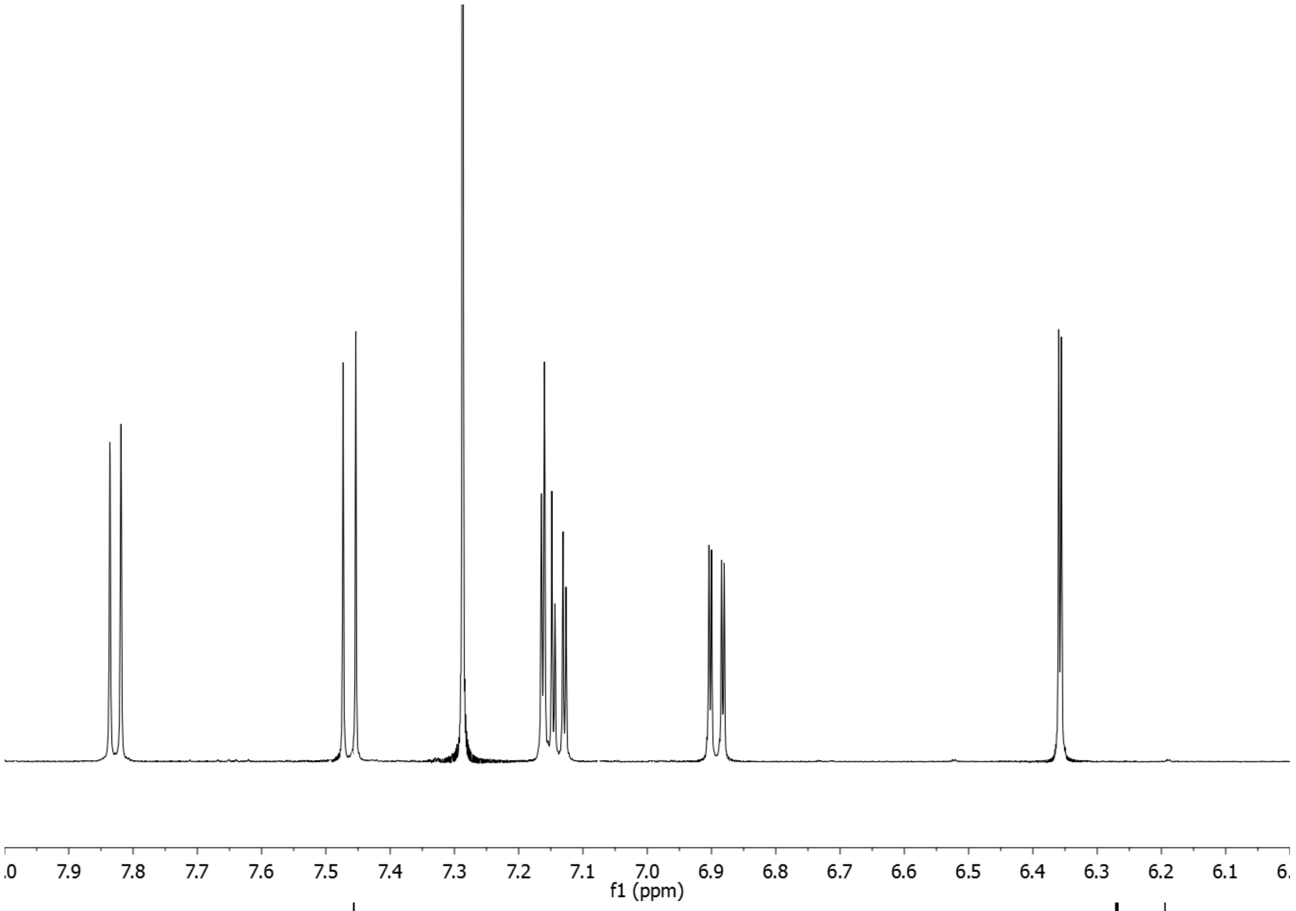

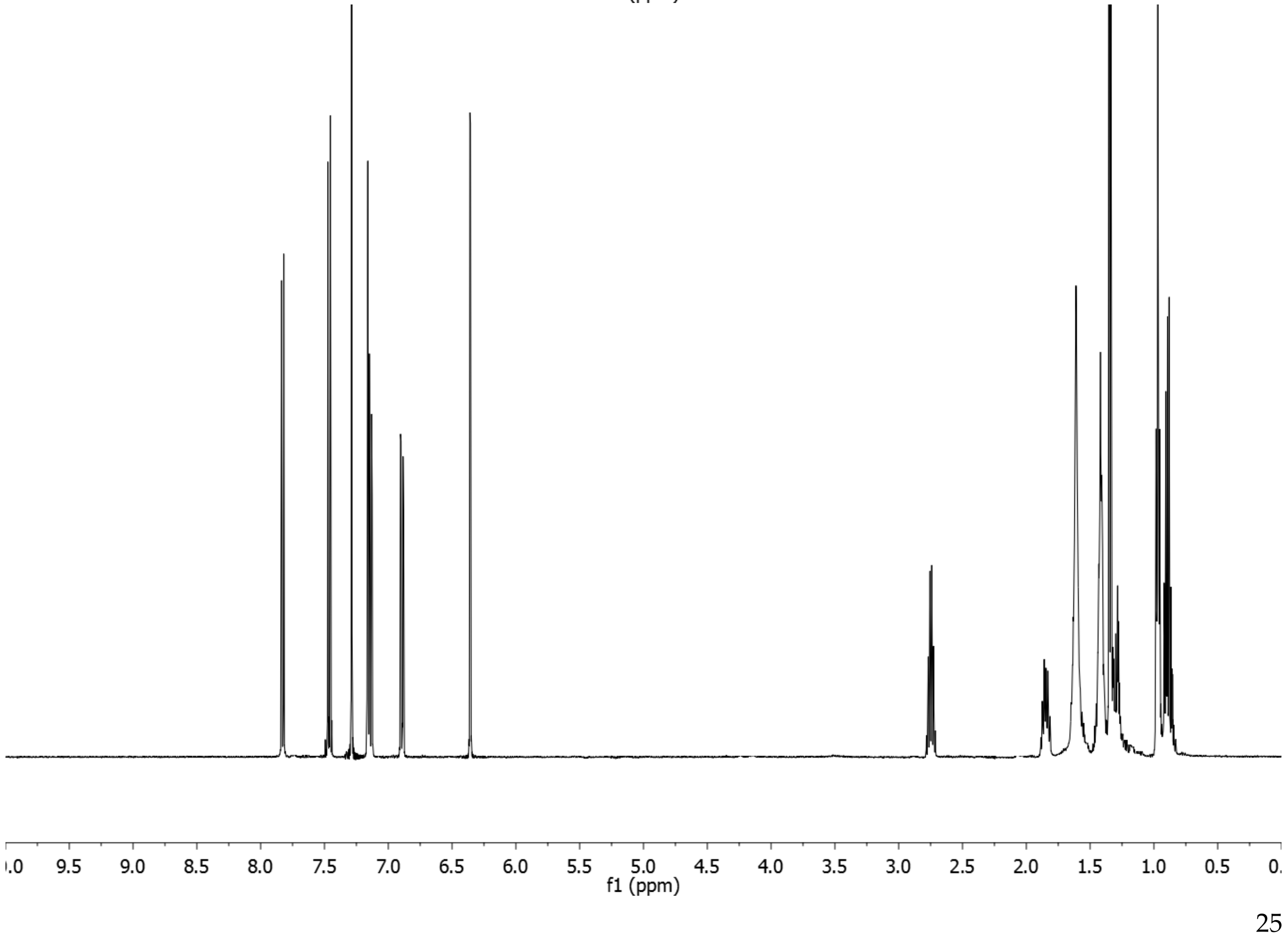

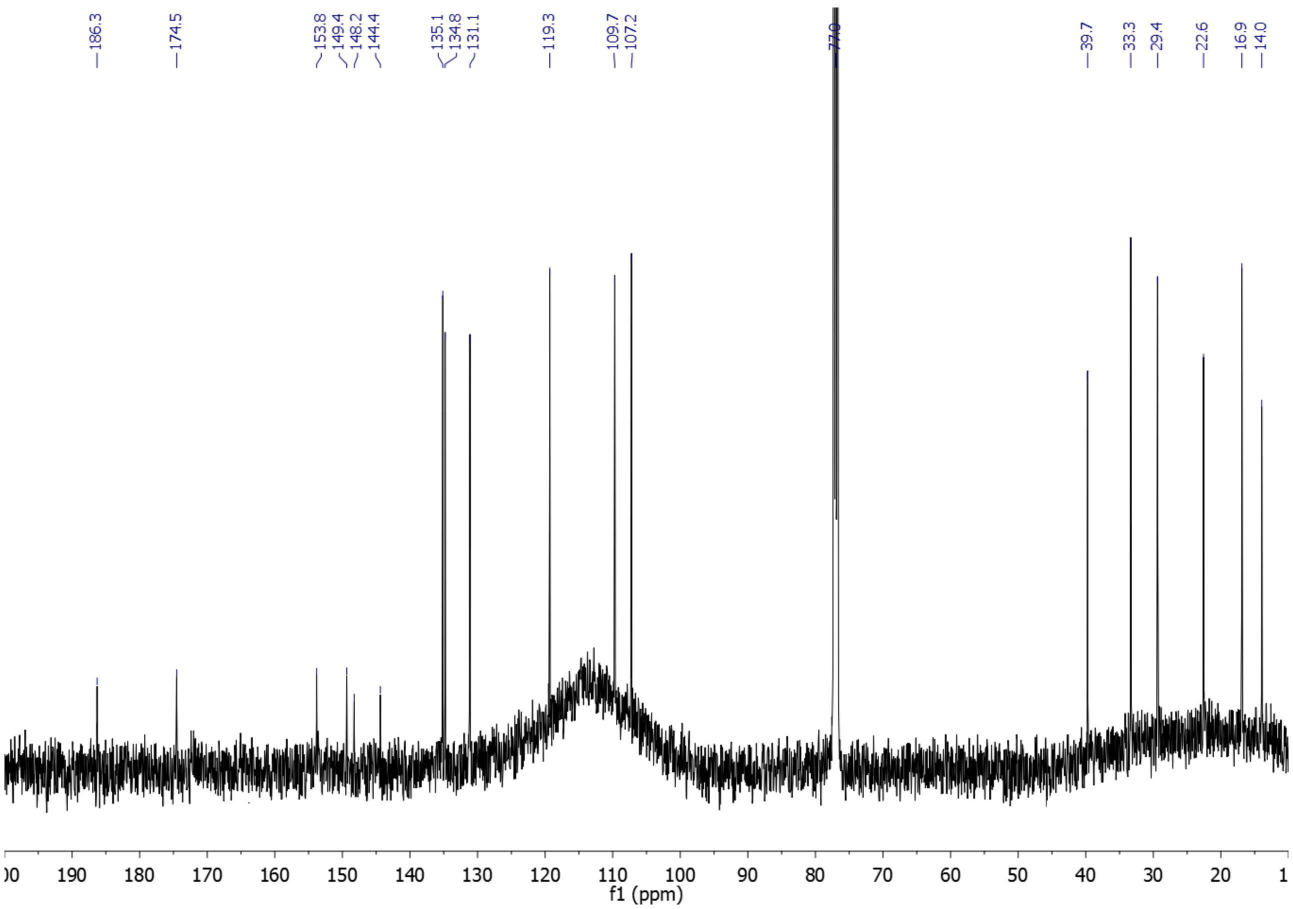
186.3
174.5
153.8
149.4
148.2
144.4
135.1
134.8
131.1
119.3
109.7
107.2
77.0
39.7
33.3
29.4
22.6
16.9
14.0
f1 (ppm)

^{1}H and ^{13}C NMR spectra of 7-hydroxyresorufinyl-2-ethylhexanoate (**1i**)

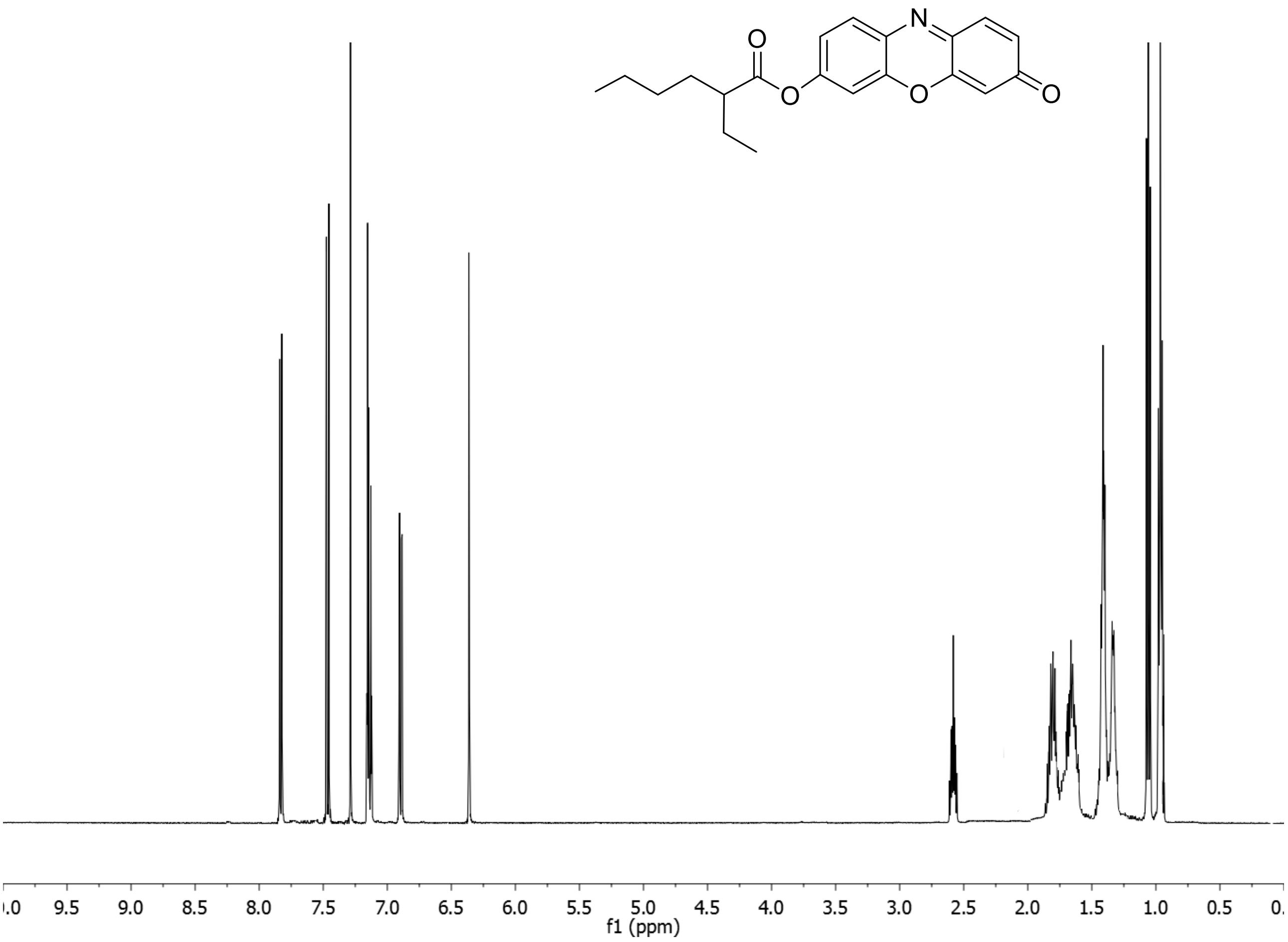

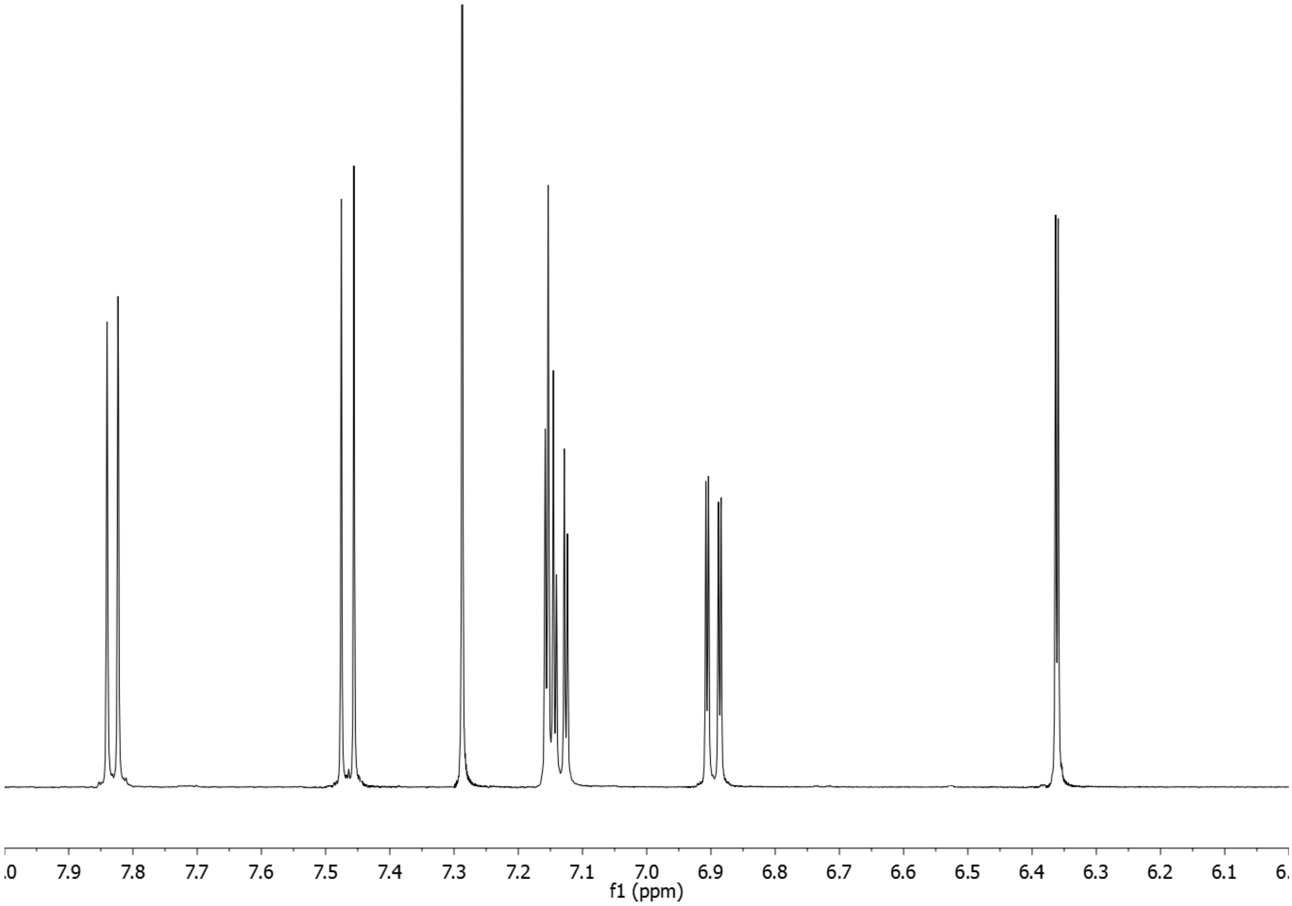

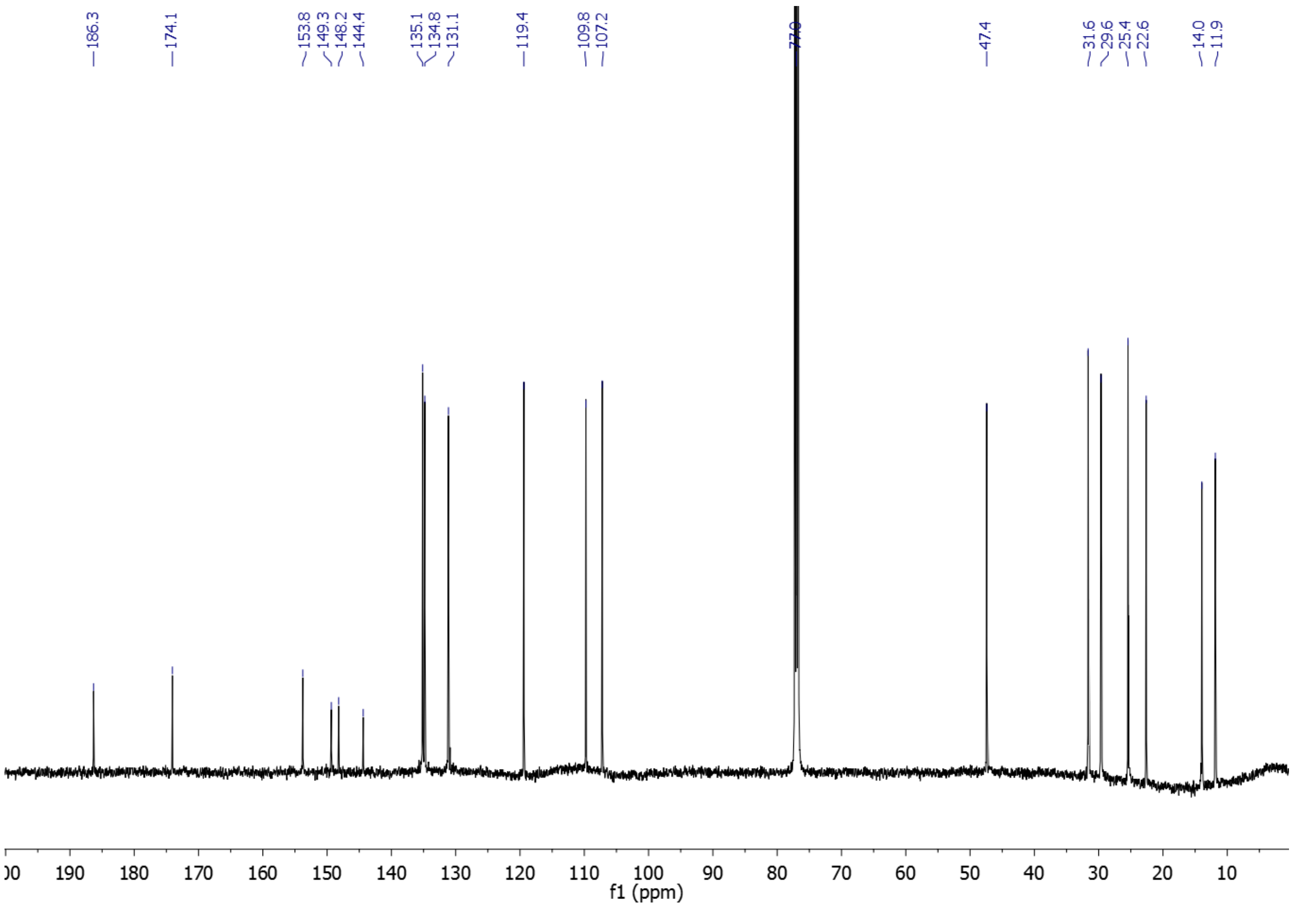
186.3
174.1
153.8
149.3
148.2
144.4
135.1
134.8
131.1
119.4
109.8
107.2
77.0
47.4
31.6
29.6
25.4
22.6
14.0
11.9
00 190 180 170 160 150 140 130 120 110 100 90 80 70 60 50 40 30 20 10
f1 (ppm)

^{1}H and ^{13}C NMR spectra
of 7-hydroxyresorufinyl-2-butyloctanoate (**1j**)

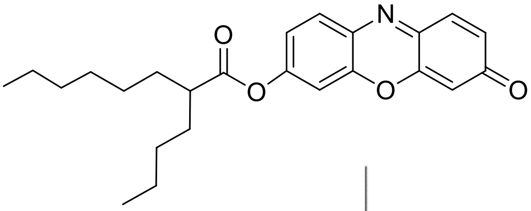

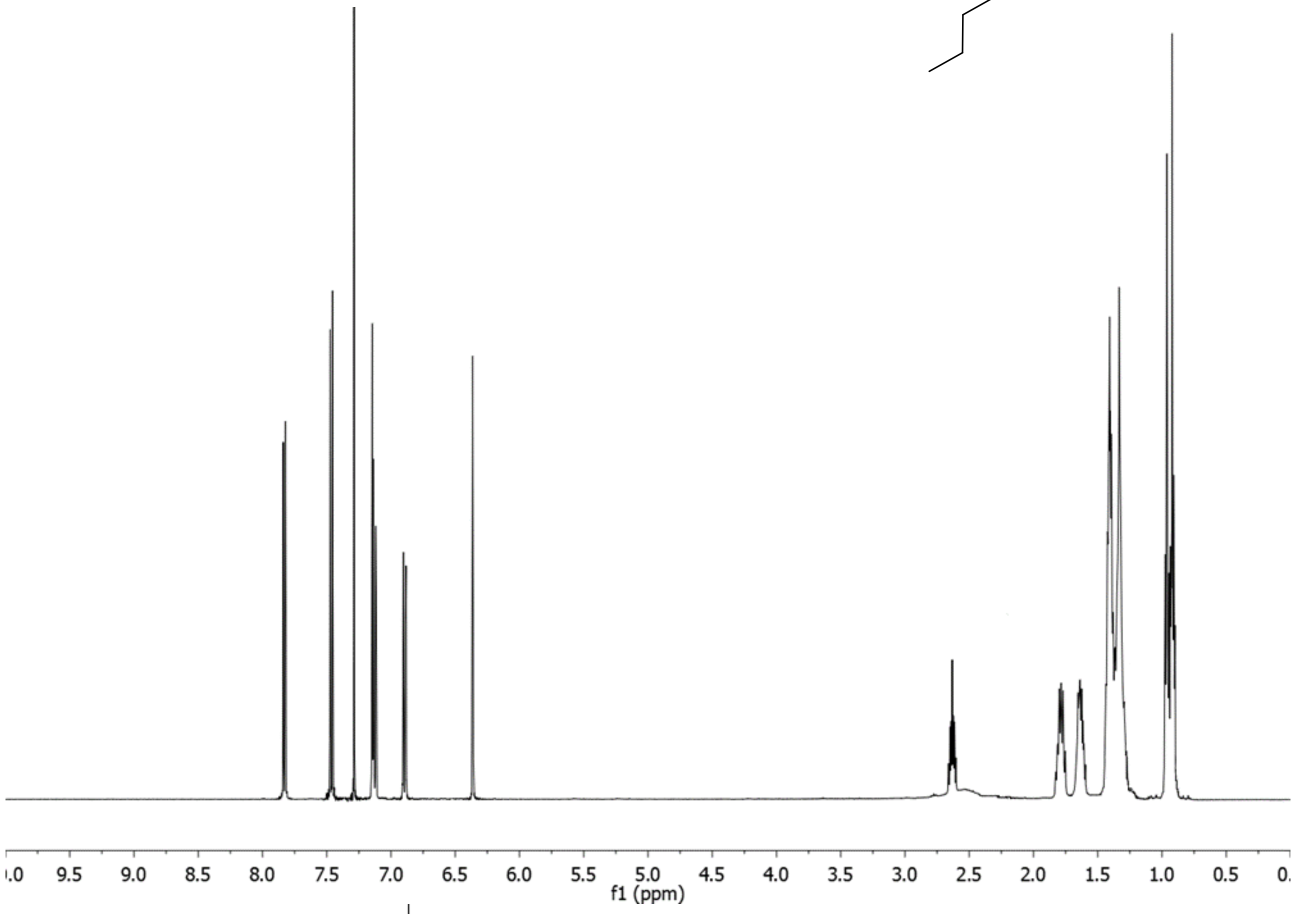

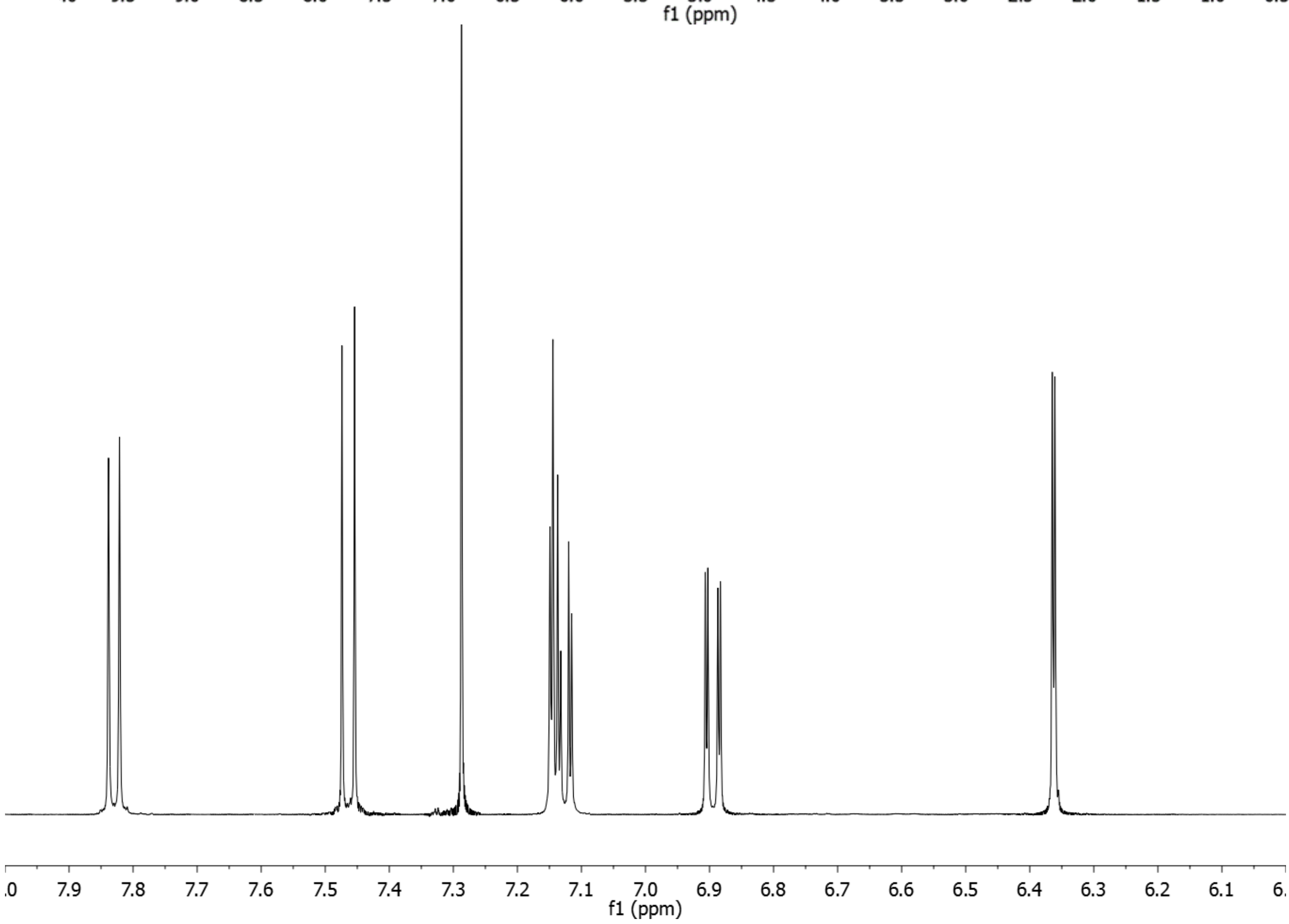

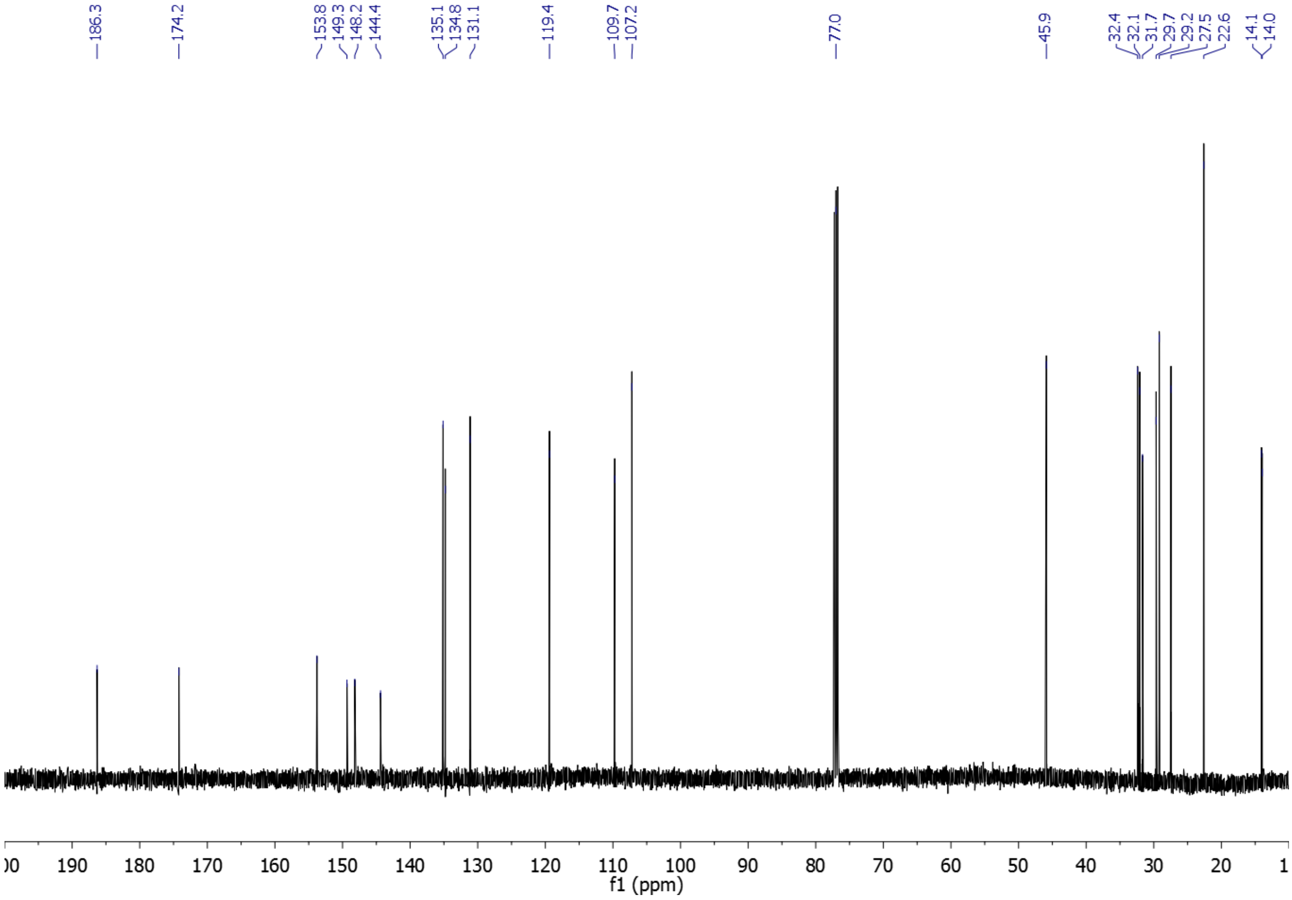
186.3
174.2
153.8
149.3
148.2
144.4
135.1
134.8
131.1
119.4
109.7
107.2
77.0
45.9
32.4
32.1
31.7
29.7
29.2
27.5
22.6
14.1
14.0
f1 (ppm)

^{1}H and ^{13}C NMR spectra of 7-hydroxyresorufinyl-2-benzoate (**1k**)

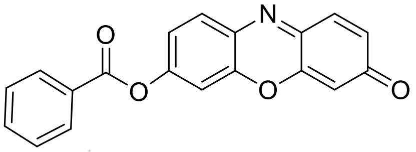

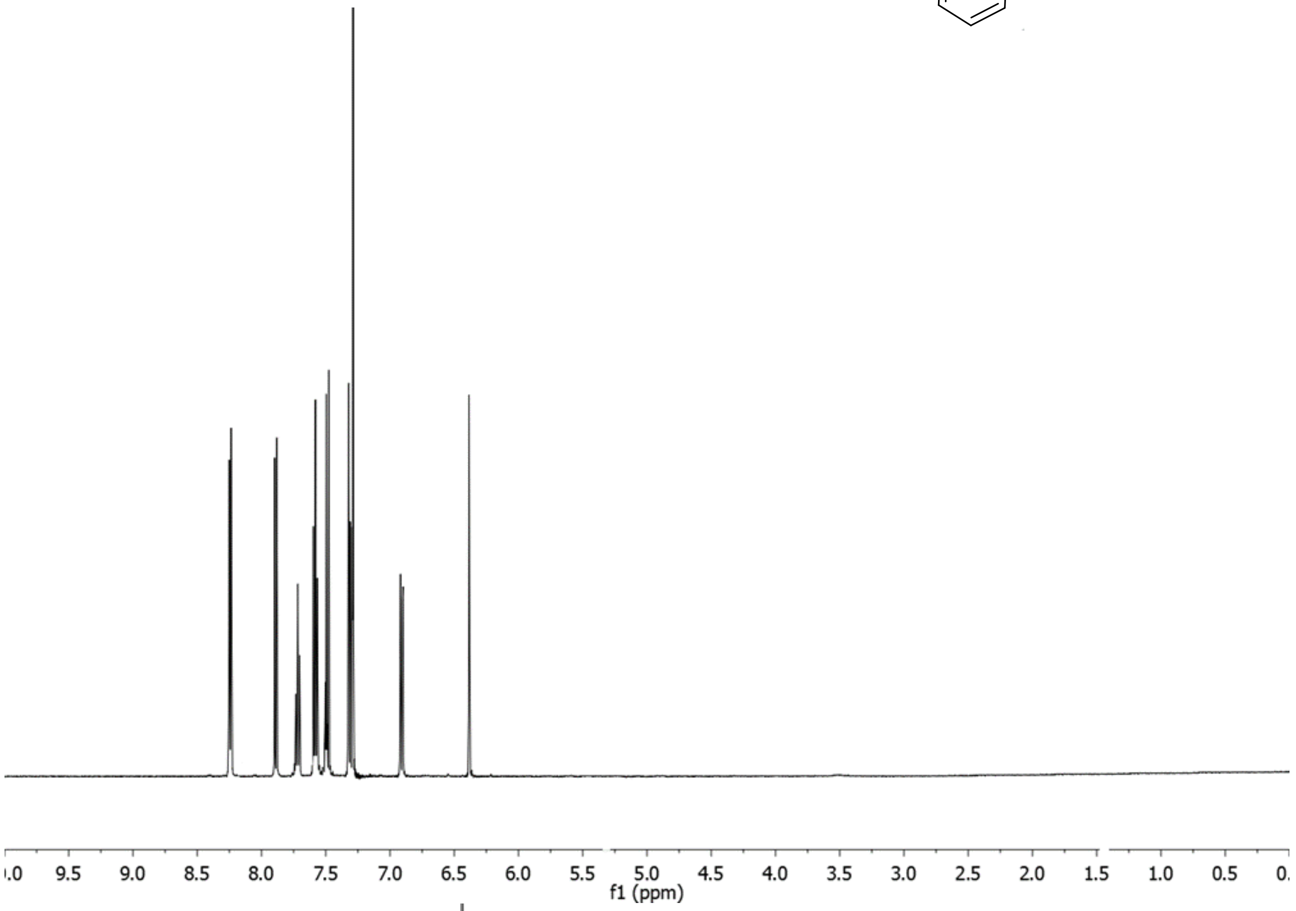

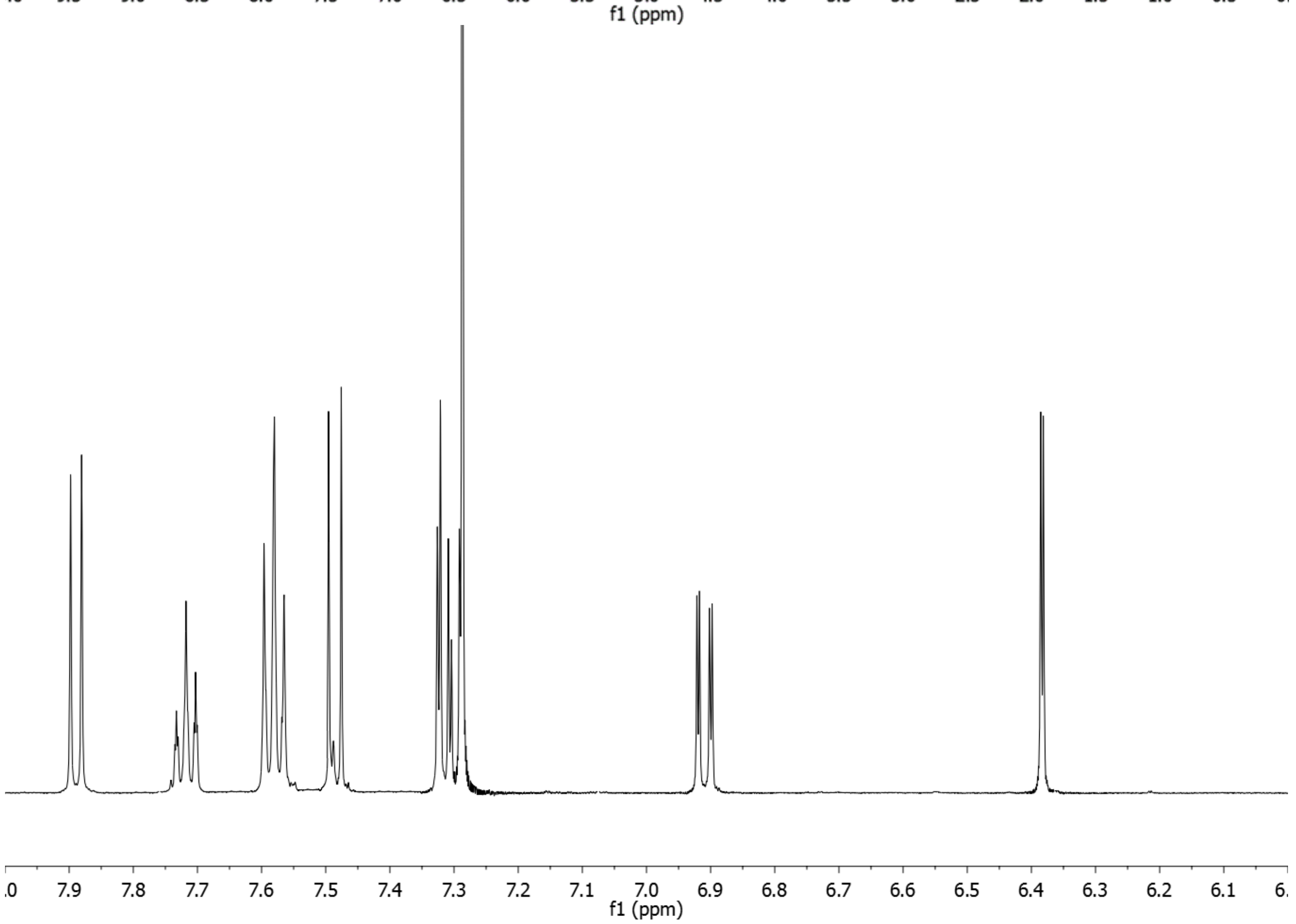

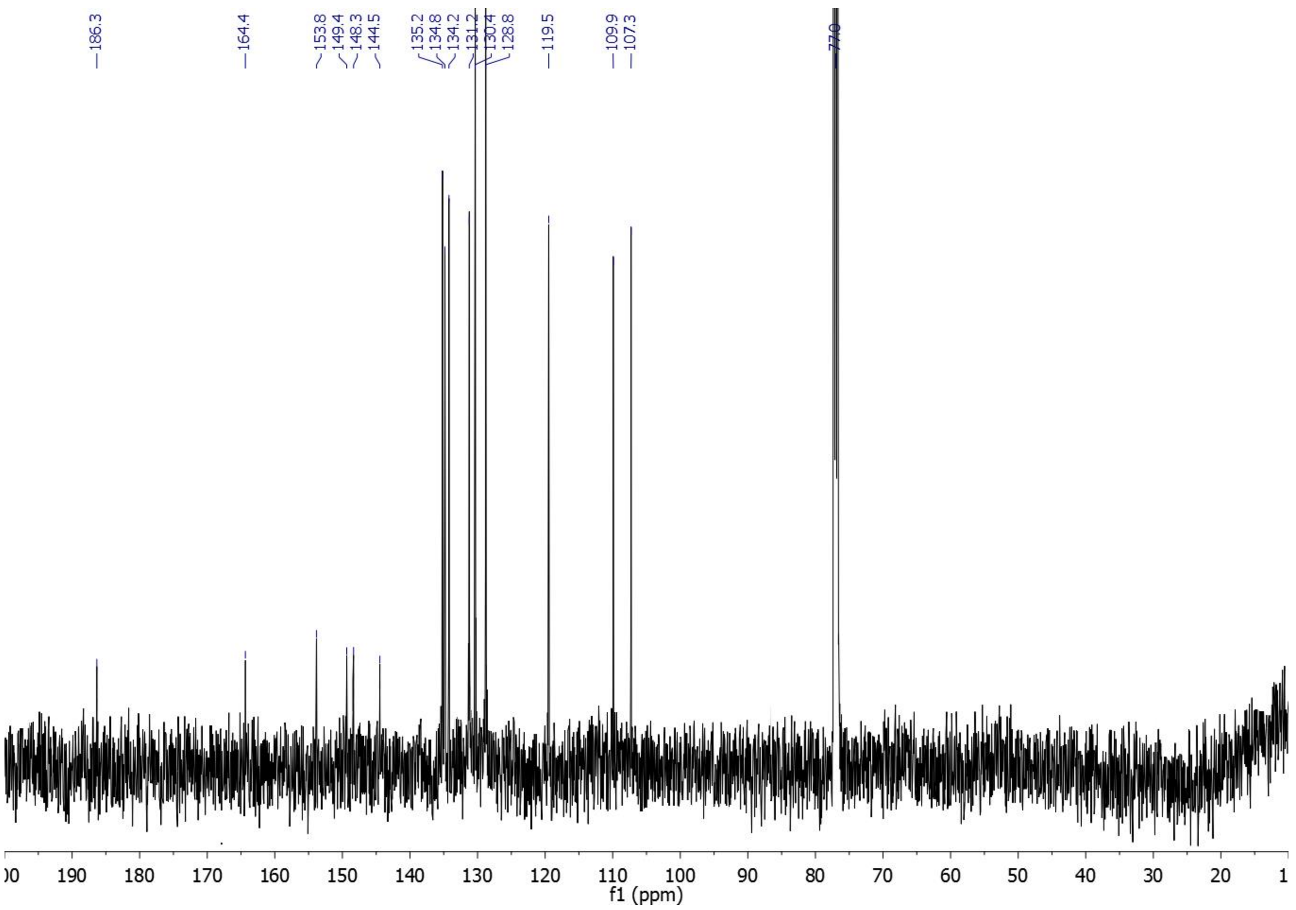
186.3
164.4
153.8
149.4
148.3
144.5
135.2
134.8
134.2
131.2
130.4
128.8
119.5
109.9
107.3
77.0
f1 (ppm)
